# *TCTEX1D2* mutations underlie Jeune asphyxiating thoracic dystrophy with impaired retrograde intraflagellar transport

**DOI:** 10.1038/ncomms8074

**Published:** 2015-06-05

**Authors:** Miriam Schmidts, Yuqing Hou, Claudio R. Cortés, Dorus A. Mans, Celine Huber, Karsten Boldt, Mitali Patel, Jeroen van Reeuwijk, Jean-Marc Plaza, Sylvia E. C. van Beersum, Zhi Min Yap, Stef J. F. Letteboer, S. Paige Taylor, Warren Herridge, Colin A. Johnson, Peter J. Scambler, Marius Ueffing, Hulya Kayserili, Deborah Krakow, Stephen M. King, Philip L. Beales, Lihadh Al-Gazali, Carol Wicking, Valerie Cormier-Daire, Ronald Roepman, Hannah M. Mitchison, George B. Witman, UK10K, Saeed Al-Turki, Carl Anderson, Richard Anney, Dinu Antony, Jennifer Asimit, Mohammad Ayub, Jeff Barrett, Inês Barroso, Jamie Bentham, Shoumo Bhattacharya, Douglas Blackwood, Martin Bobrow, Elena Bochukova, Patrick Bolton, Chris Boustred, Gerome Breen, Marie-Jo Brion, Andrew Brown, Mattia Calissano, Keren Carss, Krishna Chatterjee, Lu Chen, Sebhattin Cirak, Peter Clapham, Gail Clement, Guy Coates, David Collier, Catherine Cosgrove, Tony Cox, Nick Craddock, Lucy Crooks, Sarah Curran, Allan Daly, Petr Danecek, George Davey Smith, Aaron Day-Williams, Ian Day, Richard Durbin, Sarah Edkins, Peter Ellis, David Evans, I. Sadaf Farooqi, Ghazaleh Fatemifar, David Fitzpatrick, Paul Flicek, Jamie Floyd, A. Reghan Foley, Chris Franklin, Marta Futema, Louise Gallagher, Tom Gaunt, Daniel Geschwind, Celia Greenwood, Detelina Grozeva, Xiaosen Guo, Hugh Gurling, Deborah Hart, Audrey Hendricks, Peter Holmans, Jie Huang, Steve E. Humphries, Matt Hurles, Pirro Hysi, David Jackson, Yalda Jamshidi, David Jewell, Joyce Chris, Jane Kaye, Thomas Keane, John Kemp, Karen Kennedy, Alastair Kent, Anja Kolb-Kokocinski, Genevieve Lachance, Cordelia Langford, Irene Lee, Rui Li, Yingrui Li, Liu Ryan, Jouko Lönnqvist, Margarida Lopes, Daniel G. MacArthur, Mangino Massimo, Jonathan Marchini, John Maslen, Shane McCarthy, Peter McGuffin, Andrew McIntosh, Andrew McKechanie, Andrew McQuillin, Yasin Memari, Sarah Metrustry, Josine Min, Alireza Moayyeri, James Morris, Dawn Muddyman, Francesco Muntoni, Kate Northstone, Michael O'Donovan, Stephen O'Rahilly, Alexandros Onoufriadis, Karim Oualkacha, Michael Owen, Aarno Palotie, Kalliope Panoutsopoulou, Victoria Parker, Jeremy Parr, Lavinia Paternoster, Tiina Paunio, Felicity Payne, John Perry, Olli Pietilainen, Vincent Plagnol, Michael A. Quail, Lydia Quaye, Lucy Raymond, Karola Rehnström, J. Brent Richards, Sue Ring, Graham R S Ritchie, David B. Savage, Nadia Schoenmakers, Robert K. Semple, Eva Serra, Hashem Shihab, So-Youn Shin, David Skuse, Kerrin Small, Carol Smee, Artigas María Soler, Nicole Soranzo, Lorraine Southam, Tim Spector, Beate St Pourcain, David St. Clair, Jim Stalker, Gabriela Surdulescu, Jaana Suvisaari, Ioanna Tachmazidou, Jing Tian, Nic Timpson, Martin Tobin, Ana Valdes, Margriet van Kogelenberg, Parthiban Vijayarangakannan, Louise Wain, Klaudia Walter, Jun Wang, Kirsten Ward, Ellie Wheeler, Ros Whittall, Hywel Williams, Kathy Williamson, Scott G. Wilson, Kim Wong, Tamieka Whyte, Xu ChangJiang, Eleftheria Zeggini, Feng Zhang, Hou-Feng Zheng

**Affiliations:** 1 Genetics and Genomic Medicine Programme, University College London (UCL), Institute of Child Health, 30 Guilford Street, London WC1N 1EH, UK; 2 Department of Human Genetics, Radboud University Medical Center, 6525 GA Nijmegen, The Netherlands; 3 Radboud Institute for Molecular Life Sciences, Radboud University Medical Center, 6525 GA Nijmegen, The Netherlands; 4 Center for Pediatrics and Adolescent Medicine, University Hospital Freiburg, Mathildenstrasse 1, 79112 Freiburg, Germany; 5 Department of Cell and Developmental Biology, University of Massachusetts Medical School, Worcester, Massachusetts 01655, USA; 6 Institute for Molecular Bioscience, The University of Queensland, St Lucia, Queensland 4072, Australia; 7 INSERM UMR_1163, Département de génétique, Institut Imagine, Université Paris Descartes Sorbonne Paris Cité, Hôpital Necker-Enfants Malades, Assistance Publique-Hôpitaux de Paris, Paris 75015, France; 8 Division of Experimental Ophthalmology and Medical Proteome Center, Center of Ophthalmology, University of Tübingen, Tübingen 72074, Germany; 9 Plateforme de Bioinformatique, Institut Imagine, Université Paris Descartes, Paris 75015, France; 10 Departments of Orthopaedic Surgery and Human Genetics, David Geffen School of Medicine at UCLA, Los Angeles 90095, California, USA; 11 Section of Ophthalmology and Neuroscience, Leeds Institutes of Molecular Medicine, University of Leeds, Leeds LS9 7TF, UK; 12 Developmental Biology and Cancer Programme, University College London (UCL), Institute of Child Health, 30 Guilford Street, London WC1N 1EH, UK; 13 Medical Genetics Department, Istanbul Medical Faculty, Istanbul University, 34093 Istanbul, Turkey; 14 Medical Genetics Department, Koc University School of Medicine, 34010 Istanbul, Turkey; 15 Department of Molecular Biology and Biophysics and Institute for Systems Genomics, University of Connecticut Health Center, Farmington, Connecticut 06030, USA; 16 Centre for Translational Genomics-GOSgene, UCL Institute of Child Health, London WC1N 1EH, UK; 17 Department of Pediatrics, College of Medicine and Health Sciences, United Arab Emirates University, PO Box 17666, Al Ain, United Arab Emirates; 18 The Wellcome Trust Sanger Institute, Wellcome Trust Genome Campus, Hinxton CB10 1HH, Cambridge, UK; 19 Department of Pathology, King Abdulaziz Medical City, Riyadh, Saudi Arabia; 20 Department of Psychiatry, Trinity Centre for Health Sciences, St. James Hospital, James's Street, Dublin 8, Ireland; 21 Durham University School of Medicine, Pharmacy and Health, Wolfson Research Institute, Queen's Campus, Stockton-on-Tees TS17 6BH, UK; 22 Department of Cardiovascular Medicine and Wellcome Trust Centre for Human Genetics, Roosevelt Drive, Oxford OX3 7BN, UK; 23 Division of Psychiatry, The University of Edinburgh, Royal Edinburgh Hospital, Edinburgh EH10 5HF, UK; 24 Department of Medical Genetics, Cambridge Institute for Medical Research, University of Cambridge, Cambridge CB2 2XY, UK; 25 University of Cambridge Metabolic Research Laboratories, and NIHR Cambridge Biomedical Research Centre, Institute of Metabolic Science, Addenbrooke's Hospital, Cambridge CB2 0QQ, UK; 26 Institute of Psychiatry, Kings College London, 16 De Crespigny Park, London SE5 8AF, UK; 27 MRC CAiTE Centre, School of Social and Community Medicine, University of Bristol, Oakfield House, Oakfield Grove, Clifton, Bristol BS8 2BN, UK; 28 NIHR BRC for Mental Health, Institute of Psychiatry and SLaM NHS Trust, King's College London, 16 De Crespigny Park, London SE5 8AF, UK; 29 Dubowitz Neuromuscular Centre, UCL Institute of child health & Great Ormond Street Hospital, London WC1N 1EH, UK; 30 Department of Haematology, University of Cambridge, Long Road, Cambridge CB2 0PT, UK; 31 The Department of Twin Research & Genetic Epidemiology, King's College London, St Thomas' Campus, Lambeth Palace Road, London SE1 7EH, UK; 32 Social, Genetic and Developmental Psychiatry Centre, Institute of Psychiatry, King's College London, Denmark Hill, London SE5 8AF, UK; 33 Lilly Research Laboratories, Eli Lilly & Co. Ltd., Erl Wood Manor, Sunninghill Road, Windlesham, Surrey, UK; 34 MRC Centre for Neuropsychiatric Genetics & Genomics, Institute of Psychological Medicine & Clinical Neurosciences, School of Medicine, Cardiff University, Cardiff CF14 4XN; 35 Bart's and the London School of Medicine and Dentistry, East Lindon NHS Foundation Trust, London, UK; 36 Department of Translational Sciences, Biogen Idec, 14 Cambridge Center, Cambridge, MA 02142, USA; 37 MRC Human Genetics Unit, MRC Institute of Genetic and Molecular Medicine, at the University of Edinburgh, Western General Hospital, Edinburgh, EH4 2XU, UK; 38 European Bioinformatics Institute, Wellcome Trust Genome Campus, Hinxton, Cambridge, CB10 1SD, UK; 39 Cardiovascular Genetics, BHF Laboratories, Rayne Building, Institute Cardiovascular Sciences, University College London, London WC1E 6JJ, UK; 40 UCLA David Geffen School of Medicine, Los Angeles, California, USA; 41 Departments of Epidemiology, Biostatistics and Occupational Health, Lady Davis Institute, Jewish General Hospital, McGill University, Montreal, Quebec, Canada; 42 Department of Oncology, McGill University, Montreal, Quebec, Canada; 43 BGI-Shenzhen, Shenzhen 518083, China; 44 Molecular Psychiatry Laboratory, Mental Health Sciences Unit, University College London, 21 University St. Rockefeller Building, London WC1E 6BT, UK; 45 Human Genetics Research Centre, St George's University of London, UK; 46 HeLEX—Centre for Health, Law and Emerging Technologies, Department of Public Health, University of Oxford, Old Road Campus, Oxford, OX3 7LF, UK; 47 Genetic Alliance UK, 4D Leroy House, 436 Essex Road, London N1 3QP, UK; 48 Behavioural and Brain Sciences Unit, UCL Institute of Child Health, London, WC1N 1EH, UK; 49 Departments of Medicine & Human Genetics, Lady Davis Institute, Jewish General Hospital, McGill University, Montreal, Quebec, Canada.; 50 BGI-Europe, London; 51 National Institute for Health and Welfare (THL), Helsinki; 52 Wellcome Trust Centre for Human Genetics, Roosevelt Drive, Oxford, OX3 7BN, UK; 53 Analytic and Translational Genetics Unit, Massachusetts General Hospital, Boston MA 02113, USA; 54 Program in Medical and Population Genetics, Broad Institute of Harvard and MIT, Cambridge MA 02132, USA; 55 Department of Statistics, University of Oxford, 1 South Parks Road, Oxford OX1 3TG, UK; 56 Department of Mathematics, Université de Québec À Montréal, Montréal, Québec, Canada; 57 Institute for Molecular Medicine Finland (FIMM), University of Helsinki, Helsinki, Finland; 58 Institute of Neuroscience, Henry Wellcome Building for Neuroecology, Newcastle University, Framlington Place, Newcastle upon Tyne, NE2 4HH, UK; 59 Genetics of Complex Traits, Peninsula Medical School, University of Exeter, Exeter, UK; 60 Center for Statistical Genetics, University of Michigan, Ann Arbor, Michigan, USA; 61 University College London (UCL) Genetics Institute (UGI) Gower Street, London, WC1E 6BT, UK; 62 Departments of Health Sciences and Genetics, University of Leicester, Leicester, UK; 63 Institute of Medical Sciences, University of Aberdeen, AB25 2ZD, UK; 64 School of Medicine and Pharmacology, University of Western Australia, Perth, WA, Australia; 65 Department of Endocrinology and Diabetes, Sir Charles Gairdner Hospital, Nedlands, WA, Australia

## Abstract

The analysis of individuals with ciliary chondrodysplasias can shed light on sensitive mechanisms controlling ciliogenesis and cell signalling that are essential to embryonic development and survival. Here we identify *TCTEX1D2* mutations causing Jeune asphyxiating thoracic dystrophy with partially penetrant inheritance. Loss of TCTEX1D2 impairs retrograde intraflagellar transport (IFT) in humans and the protist *Chlamydomonas*, accompanied by destabilization of the retrograde IFT dynein motor. We thus define TCTEX1D2 as an integral component of the evolutionarily conserved retrograde IFT machinery. In complex with several IFT dynein light chains, it is required for correct vertebrate skeletal formation but may be functionally redundant under certain conditions.

## Abstract (web summary)

Severe congenital development defects such as Jeune syndrome can result from the malfunction of primary cilia and dynein. Here Schmidts *et al*. report unique biallelic null mutations in a gene encoding a dynein light chain, helping to explain the nature of ciliopathies in human patients.

## Graphical abstract

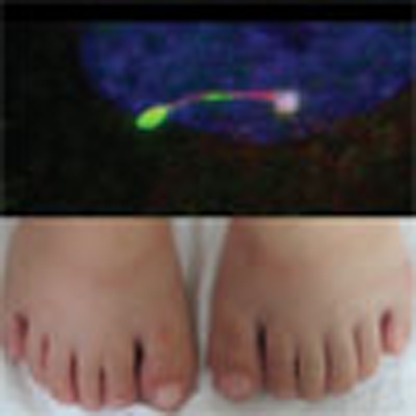

The malfunction of primary cilia, conserved signalling organelles present on the surface of most cells, has emerged as the cause of a growing number of severe congenital developmental defects^1^,^2^. Intraflagellar transport (IFT), a highly conserved process required for ciliary growth and signalling, is powered by motors attached to IFT complexes A and B. The kinesin-2 motor transports cargo along ciliary microtubules towards the ciliary tip (anterograde IFT). IFT complexes and other cargo are returned to the base of the cilium (retrograde IFT) by a specialized cytoplasmic dynein^3^, termed dynein 2 in vertebrates and dynein 1b in *Chlamydomonas*; (hereafter referred to as ‘IFT dynein'). *Chlamydomonas* has been a key organism for elucidating the molecular and mechanistic basis of IFT. The known subunits of *Chlamydomonas* IFT dynein are the homodimer-forming heavy chain DHC1b, intermediate chains D1bIC1 and D1bIC2 (also known as FAP163 and FAP133, respectively^4^), light-intermediate chain D1bLIC and the light-chain LC8. Human IFT dynein contains the homologues of these proteins, DYNC2H1, WDR60, WDR34, DYNC2LI1 and DYNLL1/DYNLL2 (both of which are LC8 homologues), and probably additional subunits^4^,^5^,^6^,^7^,^8^,^9^,^10^. However, the regulation, cargo interactions and exact composition of IFT dynein have remained relatively elusive.

Primary cilia and IFT are required for vertebrate hedgehog signalling, an important regulator of skeletogenesis^11^. Mutations in several IFT dynein components cause short-rib polydactyly syndromes (SRPS) and Jeune asphyxiating thoracic dystrophy (JATD; Jeune syndrome), a group of autosomal recessively inherited, genetically heterogeneous ciliary chondrodysplasias with overlapping phenotypic features^12^,^13^,^14^,^15^,^16^. The spectrum of disease phenotypes varies in affected individuals but hallmarks include short ribs and narrow thorax, short limbs, with sporadic polydactyly and extraskeletal disease including kidney, liver, eye, heart and brain defects. The underlying genetic basis of these skeletal ciliopathies also overlaps, with JATD at the milder end of the JATD/SRPS disease spectrum^17^. To date, mutations in 10 genes have been shown to cause Jeune syndrome, eight leading to classic JATD with ciliogenesis defects due to IFT dysfunction; these encode heavy and intermediate IFT dynein subunits DYNC2H1, WDR34 and WDR60 (refs 12, 13, 14, 15, 16), IFT complex B components IFT80 and IFT172 (refs 18, 20), and IFT complex A components WDR19/IFT144, TTC21B/IFT139 and IFT140 (refs 21, 22, 23). Two encode the centriole-associated proteins CEP120 and CSPP1 (Jeune variants), which are important for ciliary assembly or function^19^,^24^. Disease-causing mutations are considered hypomorphic since no individuals with SRPS or JATD were previously shown to carry biallelic loss-of-function mutations^13^,^17^, and homozygosity for ‘null' alleles is embryonic lethal in mouse models around midgestation^25^,^26^.

Here we report biallelic loss-of-function mutations causing JATD in the gene encoding TCTEX1D2, an IFT dynein light chain distinct from DYNLL1/DYNLL2 (LC8). Unusually for the SRPS/JATD spectrum, affected individuals all carry biallelic ‘null' alleles where complete loss of protein function is predicted. Furthermore, the disease phenotype appears incompletely penetrant. Affinity proteomics indicates that TCTEX1D2/Tctex2b is an integral component of IFT dynein. We demonstrate a retrograde IFT defect in *TCTEX1D2*-deficient human fibroblasts and Tctex2b-deficient *Chlamydomonas* cells, and find that IFT dynein is partially destabilized by loss of Tctex2b in *Chlamydomonas*. Compared with mutations in other IFT dynein components, TCTEX1D2/Tctex2b loss in human, zebrafish and *Chlamydomonas* has a modest effect on retrograde IFT, likely explaining the partially penetrant nature of human *TCTEX1D2* mutations.

## Results

### Variants in *TCTEX1D2* are associated with JATD

Whole-exome sequencing of 69 individuals from 60 families clinically diagnosed with JATD identified a homozygous consensus splice variant (c.113+2C>G) in *TCTEX1D2* (NM_152773.4, encoding a dynein light chain) in individual UCL82 II.1 from a consanguineous Turkish family (Supplementary Fig. 1a). Reverse transcription PCR (RT-PCR) on RNA derived from fibroblasts of UCL82 II.1 detected no *TCTEX1D2* transcript, suggesting nonsense-mediated decay (NMD) of the mutant transcript (Supplementary Fig. 2a). All primers used for human genetic analysis are listed in Supplementary Table 1. Exome copy number variant analysis revealed a >10-kb homozygous deletion in two affected siblings (UCL4 II.6 and II.8) from a consanguineous Arabic family that removes exon 1–2 of *TCTEX1D2* including the start codon, indicating a complete loss-of-function allele (c.(?_−142)_247+?del) (Supplementary Fig. 3). No other likely disease-causing variants in known JATD/SRPS-causing genes or other known ciliary components were detected in these two siblings. The deletion also removes exon 2–5 of neighbouring *TM4SF19* encoding a protein reportedly involved in pancreatic development but unlikely to be involved in JATD (Supplementary Fig. 3). Reverse transcription PCR on RNA derived from blood lymphocytes of individuals UCL4 II.6 and II.8 detected no *TCTEX1D2* transcript, indicating likely NMD of the mutant transcript (Supplementary Fig. 2b), and no transcripts initiated at the *TM4SF19* start codon continuing into *TCTEX1D2* downstream of the deletion either. Analysis of a further 154 JATD/SRPS exomes and Sanger sequencing of *TCTEX1D2* in 69 additional JATD/SRPS cases, previously excluded for mutations in known JATD and SRPS genes, detected compound-heterozygous variants in *TCTEX1D2* in individual INS II.1 from a non-consanguineous French family comprising a nonsense (c.262C>T; p.Arg88*) and a deletion-insertion frameshift alteration (c.100delinsCT; p.Val34Leufs*12; Supplementary Fig. 1b).

**Figure 1:**
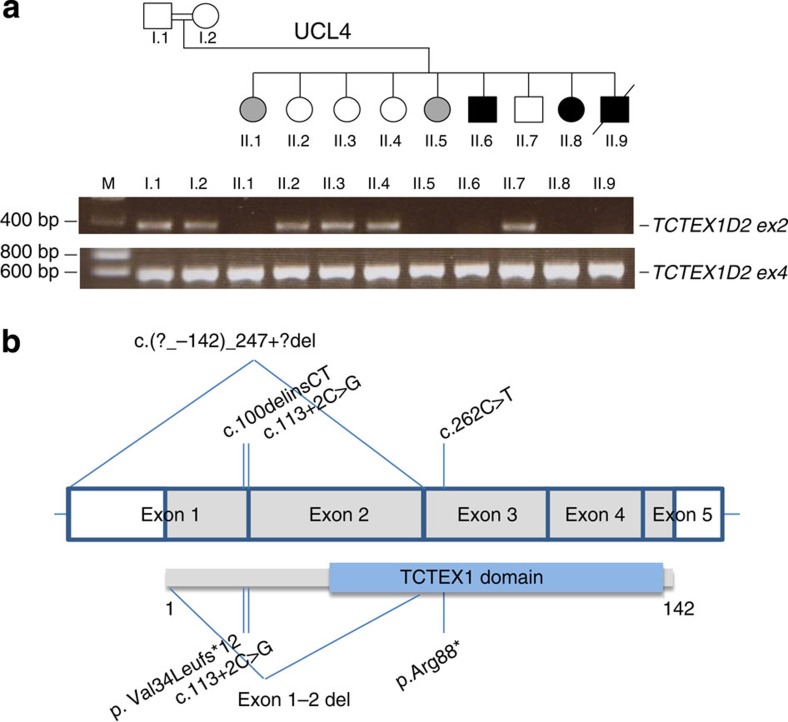
*TCTEX1D2* deletion in UCL4 and location of identified variants in TCTEX1D2 protein structure. (**a**) The absence of *TCTEX1D2* exon 1 and 2 in family UCL4 is visualized by PCR of genomic DNA samples from members of the UCL4 pedigree. *TCTEX1D2* exon 4 primers verify the presence of the gene in samples, but *TCTEX1D2* exon 2 primers do not amplify in some individuals. Children carrying the homozygous exon 1–2 *TCTEX1D2* deletion are marked in black (diagnosed with JATD) or grey (two siblings who were not diagnosed with JATD). The strikethrough indicates death at 2 months of age; double line indicates consanguineous marriage. See also Supplementary Fig. 1. (**b**) Human *TCTEX1D2* (shown above, white boxes indicate untranslated regions (UTR)) consists of five exons encoding a 142 amino-acid protein (shown below) with a C-terminal TCTEX1 domain (blue box). The location of the four identified *TCTEX1D2* mutations is shown in the gene (above). Their corresponding location in the protein (below) shows the TCTEX1 domain will be at least partially lost for all variants identified in individuals with JATD.

**Figure 2:**
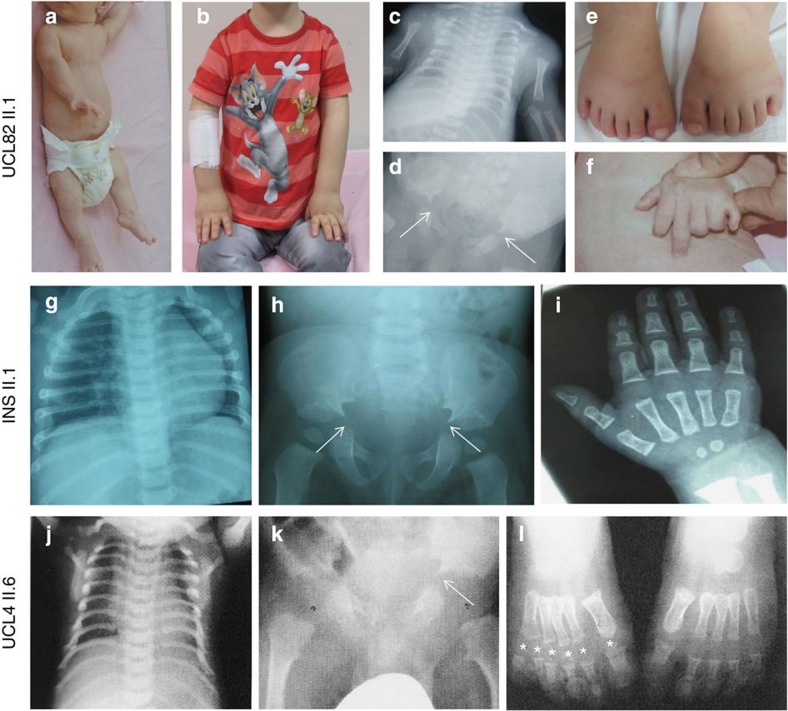
Clinical features of subjects with *TCTEX1D2* mutations. Affected individuals presented with narrow thorax due to shortened ribs (**a**–**c**, UCL82 II.1; **g**, INS family II.1; **j**, UCL4 II.6), typical pelvis configuration showing trident acetabulum with spurs (arrows) (**d**, UCL82 II.1; **h**, INS family II.1; **k**, UCL4 II.6), polydactyly (**e**,**f**, UCL82 II.1; **l**, UCL4 II.6), shortened extremities (**a**,**b**, UCL82 II.1) and brachydactyly (**i**, INS family II.1, asterisks indicate toes). UCL82 is shown at 38 days in **a**, **c**, **d** and **f**) and 5.5 yrs in **b** and **e**).

The *TCTEX1D2* c.113+2C>G, c.262C>T and c.100delinsCT variants are absent from the dbSNP, 1,000 Genomes and EVC databases. The exon 1–2 deletion is absent from 500 exomes from the UK10K project and 100 Bedouin control chromosomes assessed by Sanger sequencing. The c.113+2C>G, c.262C>T and c.100delinsCT variants segregated with the disease phenotype in affected families as expected (Supplementary Fig. 1). However, in family UCL4, the exon 1–2 deletion was detected not only in a third affected individual who died at 2 months due to respiratory insufficiency (UCL4 II.9) but also in two siblings (UCL4 II.1 and II.5) for whom no clinical signs of JATD had been documented (Fig. 1a and Supplementary Fig. 1c,d). When reassessed clinically with a full X-ray exam, both UCL4 II.1 and II.5 showed mild brachydactyly and slightly shortened lower limb distal segments; they are both shorter in stature than their siblings that do not carry the deletion, and one also had pectus carinatum as a child. However, in contrast to the other five individuals documented here as affected with typical clinical features of JATD (short horizontal ribs, narrow thorax, trident acetabulum with spurs and polydactyly; Fig. 2 and Table 1), no specific radiological signs of JATD/SRPS were found in UCL4 II.1 and II.5 (Supplementary Fig. 4). Thus, while their mild brachydactyly and short stature could represent a very mild JATD phenotype, or radiological diagnostic criteria for JATD might have been fulfilled in childhood, we have no clinical or radiological proof for JATD. Brachydactyly and short stature can occur with JATD but are not specific for the condition. We therefore suggest that the phenotype is not fully penetrant in this family, with the caveat that we cannot definitively exclude an extremely mild expression of the JATD phenotype in the two seemingly unaffected individuals harbouring *TCTEX1D2* mutations.

We examined this further by single-nucleotide polymorphism-based genome-wide linkage analysis in all members of UCL4. A high penetrance recessive model coding UCL4 II.1 and II.5 as unknown identified five homozygous linked regions all with a logarithm of odds (LOD) score of 2.9, with *TCTEX1D2* in the second largest interval (Supplementary Table 2). Cross-reference to the exome-sequencing data detected the *TCTEX1D2* deletion and just one other homozygous variant shared between UCL4 II.6 and II.8 in these intervals. The latter was in an untranslated pseudogene *TBC1D3P2* with a minor allele frequency of 1 in 500 in our in-house control database, suggesting it is not causative for JATD. Considering UCL4 II.1 and II.5 as affected under a reduced penetrance model (penetrance set at 0.6) generated three linked regions including one with a LOD score 3.9 across *TCTEX1D2*, with no homozygous variants shared between UCL4 II.6 and II.8 in the other intervals (Supplementary Table 3). Considering UCL4 II.1 and II.5 as unaffected found no linkage to homozygous variants shared between UCL4 II.6 and II.8 except the *TBC1D3P2* pseudogene variant (LOD score 2.9; Supplementary Table 4). Finally, considering UCL4 II.1 and II.5 as affected under the high penetrance model identified just one linked region, a 9.4-Mb locus containing *TCTEX1D2* with a LOD score of 4.1 (Supplementary Table 5). Thus, along with the expected segregation in two independent families, linkage modelling supports *TCTEX1D2* mutations as disease-causing in family UCL4.

Functional predictions and evidence of NMD suggest that all four *TCTEX1D2* variants identified are loss-of-function mutations affecting the conserved TCTEX1 domain of the protein (Fig. 1b). The questionable disease status in individuals UCL4 II.1 and II.5 suggests incomplete penetrance in this family, an inheritance pattern not previously described for JATD.

### Knockdown of *tctex1d2* causes a typical ciliopathy phenotype

TCTEX1D2 function was not previously investigated in vertebrates and we tested for potential redundancy of the orthologous gene in zebrafish using oligonucleotide antisense morpholinos to abrogate transcription. Morpholino oligomers have been used widely to provide models for Jeune syndrome by transiently knocking down the expression of the orthologous zebrafish genes^18^,^19^,^20^. This approach has been criticized for its inability to reliably discriminate between specific and non-specific effects such as developmental delay and cardiac oedema^27^,^28^. To increase reliability, we established that human (NP_689986.2) and zebrafish (XP_685487.3) TCTEX1D2 proteins are highly homologous, being 60% identical at 80% BLAST coverage. We separately tested two different splice-blocking morpholinos and focussed on phenotypic features described in JATD zebrafish mutants as well as morphants^29^,^30^,^31^,^32^,^33^.

**Figure 3:**
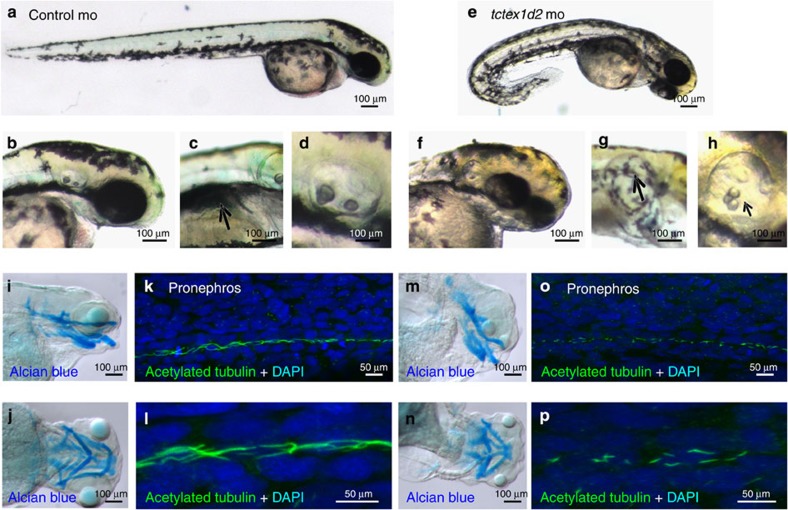
Knockdown of *tctex1d2* in zebrafish leads to a typical ciliopathy phenotype. Whole-mount light microscopy showing control morpholino (mo)-injected embryos (**a**–**d**) and *tctex1d2* morphants at 4 days post fertilization (**e**–**h**). Compared with controls, knockdown of *tctex1d2* results in ventrally curved body axis (**a**,**e**), small eyes **(b**,**f**), pronephric cysts (**c**,**g**) and otolith defects (**d**,**h**). Alcian blue staining of cartilage identifies craniofacial cartilage defects in *tctexd2* morphants (**m**,**n**) compared with controls (**i**,**j**). Immunofluorescence analysis after staining of cilia at 24 h.p.f. with anti-acetylated tubulin antibody reveals shorter cilia in the pronephric duct of *tctex1d2* morphants (**o**, magnified in **p**) compared with control embryos (**k**, magnified in **l**); however, this difference was no longer evident at 48 h.p.f. (data not shown). Scale bars, 100 μm (**a**–**j**,**m**,**n**) or 50 μm (**k**,**l**,**o**,**p**).

Both morpholinos yielded identical results, producing a dose-dependent typical ciliopathy phenotype^18^,^20^, with ventrally curved body axis, hydrocephalus, abnormal otoliths and small eyes (Fig. 3a–h). Loss of transcript was confirmed by RT–PCR (Supplementary Fig. 5 and Supplementary Table 1). The few embryos surviving to 4 days post fertilization displayed severe generalized oedema and pronephric cysts (Fig. 3c,g). Embryos also showed defects of the craniofacial cartilage as visualized by alcian blue staining (Fig. 3i,j,m,n), which were comparable to those observed in other ciliary chondrodysplasia zebrafish models such as *ift80, ift172* and *cspp1* (refs 18, 19, 20). Immunofluorescence analysis revealed shorter cilia in the pronephric duct at 24 h post fertilization (h.p.f.) in *tctex1d2* morphant embryos compared with controls (Fig. 3k,o,l,p); however, this difference was no longer observed at 48 h.p.f. (not shown). This could reflect delayed ciliogenesis in the morphants or could result from general mild developmental delay. Cilia in the neural tube appeared normal (Supplementary Fig. 6).

### Loss of *TCTEX1D2* causes a retrograde IFT defect

**Figure 4:**
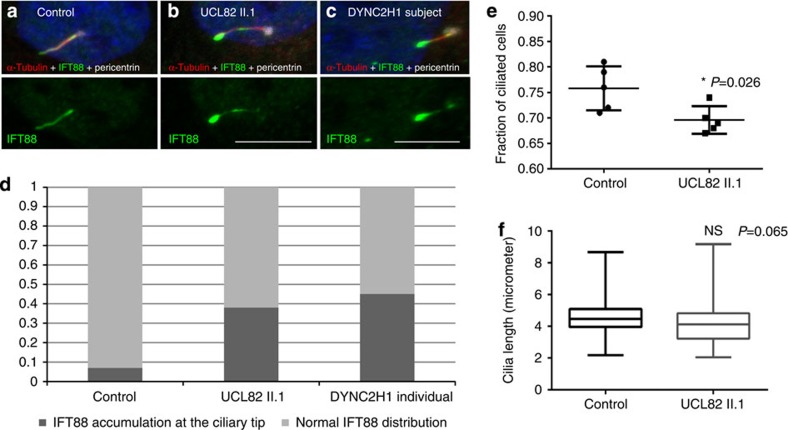
Loss of human TCTEX1D2 results in retrograde IFT defects. Immunofluorescence analysis using confocal microscopy revealed an accumulation of IFT88 at the ciliary tips in skin fibroblasts from individual UCL82 II.1 compared with a control (**a**,**b**). The accumulation is comparable to that previously reported in fibroblast cilia from an individual with JATD caused by biallelic variants in *DYNC2H1* (ref. 34) (**c**). IFT88 staining is shown in green, anti-acetylated tubulin antibody (red) was used for visualization of the ciliary axoneme, anti-pericentrin antibody (white) marks the ciliary base; the IFT88 labeling also is shown separately in the lower panels. Scale bars, 5 μm. (**d**) Fraction of cells with IFT88 accumulation at the ciliary tip, 100 cells analysed for each condition. (**e**) The percentage of ciliated cells in the fibroblast sample from individual UCL82 II.1 compared with control fibroblasts as assessed by counting the number of cilia stained with anti-acetylated tubulin antibody in relation to nuclei stained with DAPI (4,6-diamidino-2-phenylindole, blue in **a**–**c**) in 10 random visual fields in five independent experiments each, revealing a very mild reduction in ciliation for the *TCTEX1D2*-deficient cells. One hundred cells were counted per experiment, represented by a single point per experiment. (**f**) No difference in cilia length between UCL82 II.1 and control fibroblasts was visualized using anti-acetylated tubulin antibody, 100 cells analysed for each condition. Statistical significance in **e** and **f** was measured using the Student's *t*-test, asterisk indicates *P* value <0.05.

We proceeded to investigate skin fibroblasts from individual UCL82 II.1 for defects in cilia architecture and IFT disturbance. While the percentage of ciliated cells was modest but significantly lower for *TCTEX1D2* mutant fibroblasts compared with controls (Fig. 4e), no significant difference in ciliary length was observed after 24-h serum starvation, in contrast to the cilia shortening reported in individuals with mutations in other JATD/SRPS genes^12^,^19^ (Fig. 4f). However, there was marked accumulation of the IFT-particle protein IFT88 in ∼35% of the ciliary tips of *TCTEX1D2*-deficient fibroblasts, compared with <10% of control fibroblasts (Fig. 4a–d). Cells from an individual previously reported to carry *DYNC2H1* mutations^34^ showed a very similar pattern, which indicates a retrograde IFT defect (Fig. 4d).

### *Chlamydomonas* Tctex2b is a light chain of IFT dynein

We further investigated the role in IFT of Tctex2b, the *Chlamydomonas* homologue of TCTEX1D2 (49% identical at 80% BLAST coverage; human to *Chlamydomonas* reciprocal best match BLAST 9e−36). To determine whether Tctex2b is a subunit of IFT dynein in *Chlamydomonas*, we used a new strain expressing D1bIC2 (homologue of human IFT dynein intermediate chain WDR34) fused to the haemagglutin-epitope tag (HA). This was generated by transforming an insertional mutant defective for D1bIC2 (*dic5-1*; Hou *et al*., manuscript in preparation) with C-terminal HA-tagged D1bIC2. *dic5-1* cells have a severe flagellar assembly defect that is fully rescued by HA-tagged D1bIC2 (Supplementary Fig. 7), indicating its functional incorporation into IFT dynein. D1bIC2-HA has a normal distribution in the cell, is expressed in flagella at approximately normal levels and sediments normally as part of a complex when the flagellar membrane-plus-matrix is fractionated on sucrose gradients (Supplementary Fig. 7). Therefore, D1bIC2-HA behaves like wild-type D1bIC2 at all levels examined.

**Figure 5:**
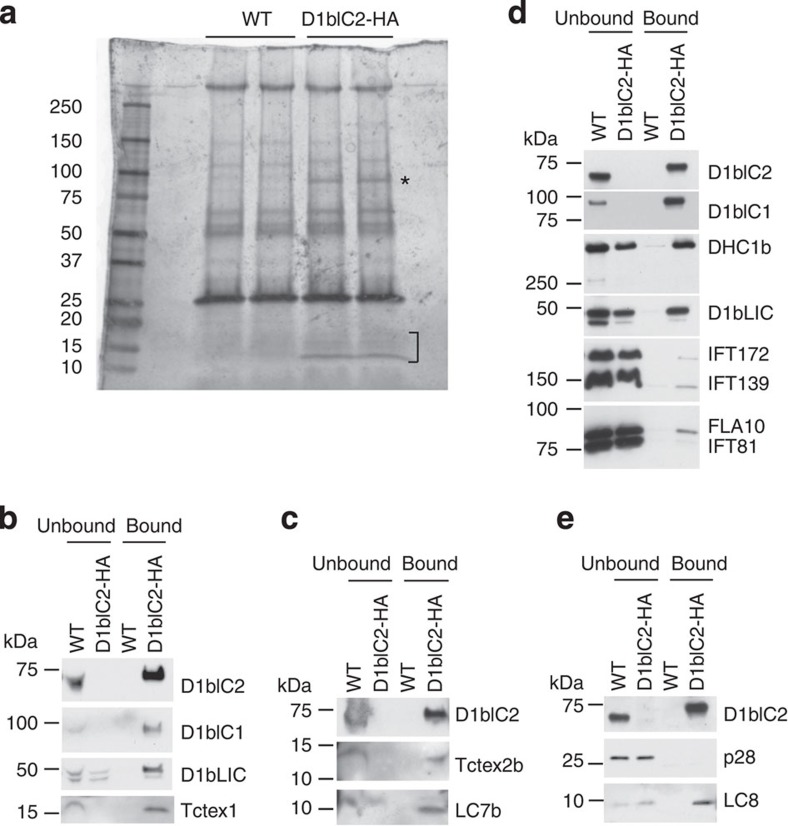
Tctex2b and Tctex1 are in an IFT dynein intermediate chain/light-chain subcomplex with D1bIC2 and D1bIC1. (**a**) Flagellar membrane-plus-matrix fractions from wild-type (WT) cells or cells expressing D1bIC2-HA were incubated with anti-HA antibody-conjugated beads. The proteins pulled down by the beads were separated by SDS–PAGE and silver stained. One band (marked *) between 75 and 100 kDa and several bands around 15 kDa (marked ]) are specific for the D1bIC2-HA sample. (**b**–**d**) Western blots confirming that Tctex2b, Tctex1 and D1bIC1 are specifically co-precipitated with D1bIC2-HA. The unbound and bound samples were probed with the indicated antibodies. In each experiment, all of the D1bIC2-HA was immunoprecipitated from the D1bIC2-HA sample; all of the D1bIC1 (**b**,**d**), Tctex1 (**b**) and Tctex2b (**c**) was co-precipitated from the D1bIC2-HA sample. None of these proteins were pulled down from the WT control. Some but not all of the DHC1b (**d**), D1bLIC (**b**,**d**), LC7b (**c**) and LC8 (**e**) was co-precipitated from the D1bIC2-HA samples; (**d**) also shows that only very small amounts of the IFT-particle proteins or FLA10 were co-precipitated with D1bIC2-HA. (**e**) Similar western blot showing that p28 was not co-precipitated with D1bIC2-HA. In **b**, **d** and **e**, the ratio of unbound: bound protein loaded was 1:4; in **c**, the ratio was 1:2. All antibodies used for *Chlamydomonas* protein analysis are listed in Supplementary Table 6.

We used anti-HA antibody-conjugated beads to immunoprecipitate D1bIC2-HA protein from the membrane-plus-matrix fraction of isolated steady-state (that is, non-assembling) flagella of the D1bIC2-HA strain. SDS–polyacrylamide gel electrophoresis (SDS–PAGE) detected bands at ∼*M*_r_ 90,000 and 15,000 in the D1bIC2-HA immunoprecipitate that were absent from the wild-type control (Fig. 5a). Mass spectrometry of excised bands identified several proteins specific for the D1bIC2-HA sample, including D1bIC1 (homologue of *H.s*. WDR60), Tctex2b, Tctex1 (homologous to both *H.s*. DYNLT1 and DYNLT3) and LC8 (homologous to both *H.s*. DYNLL1 and DYNLL2). LC7a (homologue of *H.s*. DYNLRB1 and DYNLRB2) was identified by nine peptides in the D1bIC2-HA sample and two peptides in the wild-type control. Western blotting confirmed that D1bIC1, Tctex2b, Tctex1 and LC8 were specific for the D1bIC2-HA sample (Fig. 5b–e), and also identified LC7b (also a homologue of *H.s*. DYNLRB1 and DYNLRB2) whose peptides were not detected in the immunoprecipitate because it migrated below the excised slice (Fig. 5c). Moreover, all detectable D1bIC1, Tctex2b and Tctex1 were co-immunoprecipitated from the fraction (Fig. 5b–d). About one-half of the LC7b, about one-quarter of the DHC1b, D1bLIC and LC8, and trace amounts of FLA10 (a kinesin-2 heavy chain) and several IFT-particle proteins also were co-immunoprecipitated with D1bIC2 (Fig. 5b–e).

Tctex2b and Tctex1 have not previously been shown to be associated with IFT dynein in *Chlamydomonas*, but they are known subunits of the axonemal inner arm dynein I1/f, which is involved in flagellar motility^35^. Dynein I1/f is present at very low levels in the membrane-plus-matrix fraction of steady-state flagella^36^. Nevertheless, to be sure that the presence of Tctex2b and Tctex1 in the D1bIC2-HA immunoprecipitate was not due to contamination by dynein I1/f, we probed blots of the unbound and bound membrane-plus-matrix with an antibody to IC140, an I1/f intermediate chain^37^, and found no IC140 in either the unbound or bound fractions of either the wild-type or mutant extracts. We also probed for p28, a subunit common to several other inner arm dyneins and previously found in the membrane-plus-matrix fraction of steady-state flagella^36^. We readily detected p28 in the unbound fractions, but not in the bound fractions, indicating that the bound fractions were not contaminated by these other inner arm dyneins (Fig. 5e). We conclude that Tctex2b and Tctex1 are novel components of *Chlamydomonas* IFT dynein. These results also show that the light chains Tctex2b and Tctex1, and intermediate chain D1bIC1 are more closely associated with D1bIC2 than with the heavy and light-intermediate chains DHC1b and D1bLIC. It is therefore likely that D1bIC2, D1bIC1, Tctex1, Tctex2b and LC7b together with LC8 form a discrete intermediate chain/light-chain complex within IFT dynein.

### Tctex2b is involved in retrograde IFT

**Figure 6:**
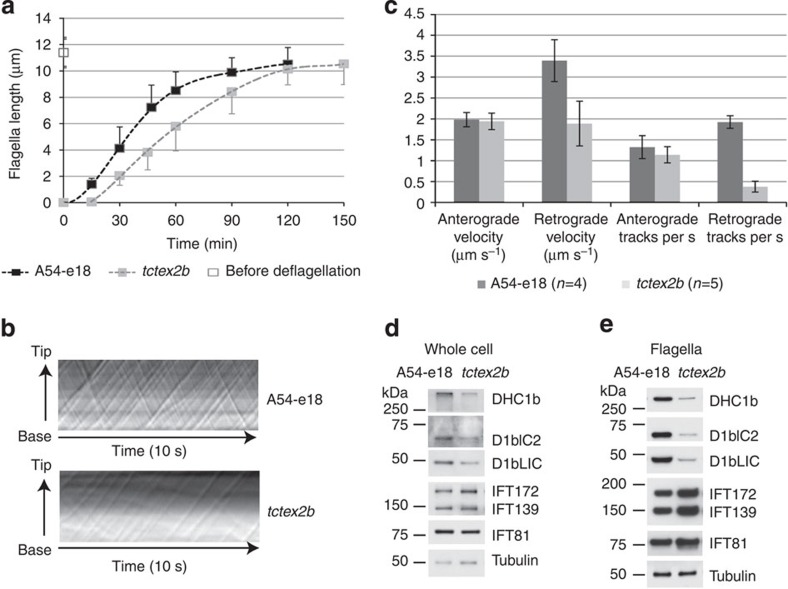
Loss of Tctex2b causes IFT dynein instability and a retrograde IFT defect in *Chlamydomonas*. (**a**) The *tctex2b* mutant has defects in flagella regeneration. The *tctex2b* null mutant and A54-e18 (the wild-type strain from which *tctex2b* was derived) were deflagellated and then allowed to regrow their flagella. Flagella lengths were measured before deflagellation and at time points after deflagellation. The two strains had identical flagellar lengths (11.4 μm) before deflagellation. For each time point, one flagellum from each of 50 cells was measured; error bars are s.d. (**b**) The *tctex2b* mutant is defective in retrograde IFT. IFT was recorded in wild-type (A54-e18) and *tctex2b* flagella by DIC microscopy, and kymograms generated from the video recordings. Tracks with positive slopes represent IFT particles moving anterogradely, and tracks with negative slopes represent particles moving retrogradely. Compared with wild type, few retrograde tracks are visible in the *tctex2b* kymogram, and these had a much reduced slope. Retrograde particles had a larger apparent size in mutants; similar findings were reported for a temperature-sensitive *dhc1b* mutant^47^. (**c**) Quantitative analysis of IFT in wild type (A54-e18) and *tctex2b*. In *tctex2b*, anterograde IFT velocity is about the same as in wild type, while anterograde frequency is only slightly reduced, but both retrograde IFT velocity and frequency are greatly reduced. *n*, number of flagella analysed. Error bars show s.d. (**d**) Western blot showing reduced IFT dynein subunits in *tctex2b* whole-cell lysates. Wild-type (A54-e18) and *tctex2b* whole-cell lysates were probed with antibodies to IFT dynein subunits and IFT-particle proteins. DHC1b, D1bIC2 and D1bLIC are reduced in *tctex2b* whole-cell lysate. No significant changes were detected for IFT proteins. The same samples were probed for tubulin as loading control. (**e**) IFT dynein is greatly reduced in *tctex2b* flagella. Wild-type (A54-e18) and *tctex2b* flagella were probed with antibodies to IFT dynein subunits and IFT-particle proteins. IFT dynein subunits DHC1b, D1bIC2 and D1bLIC are greatly reduced in *tctex2b* flagella. IFT-A protein IFT139 and IFT-B proteins IFT172 and IFT81 are increased in *tctex2b* flagella, consistent with a retrograde IFT defect. The same samples were probed for tubulin as loading control.

With this evidence that Tctex2b is a subunit of IFT dynein, we investigated whether loss of Tctex2b affects retrograde IFT. We re-examined a *Chlamydomonas* Tctex2b null strain (here termed *tctex2b*)^35^ previously shown to swim slower than wild-type cells owing to a defect in inner arm dynein I1/f, but not examined for defects in retrograde IFT. The *tctex2b* mutant has normal length flagella at steady state, but its rate of flagellar assembly is about one-third less than that of wild type (Fig. 6a). Differential interference contrast (DIC) microscopy revealed that anterograde IFT velocity is normal, and anterograde IFT frequency only slightly reduced (Fig. 6b,c). However, retrograde IFT velocity is ∼50% reduced, and retrograde IFT frequency is <25% that of wild type (Fig. 6b,c). Therefore, loss of Tctex2b has a severe effect specifically on retrograde IFT, indicating that Tctex2b is required for normal retrograde IFT.

### IFT dynein is destabilized in the absence of Tctex2b

To better understand why retrograde IFT frequency and velocity are reduced in the *Chlamydomonas tctex2b* null mutant, we compared IFT dynein levels in wild-type and *tctex2b* whole-cell lysates by western blotting (Fig. 6d). DHC1b, D1bIC2 and D1bLIC were each reduced by ∼40–60% in the mutant cells relative to wild type. These results strongly suggest that IFT dynein is destabilized in the absence of Tctex2b, with resulting degradation of the protein complex. In isolated *tctex2b* flagella, levels of DHC1b, D1bIC2 and D1bLIC were reduced even further (Fig. 6e) to only ∼15–25% that of wild type. This likely accounts for the reduced frequency of retrograde IFT in the mutant.

Western blotting also revealed that the mutant whole cells had normal levels of IFT-particle proteins (Fig. 6d), but the mutant flagella had increased amounts of all IFT complex A and B proteins examined (Fig. 6e). This is in agreement with the IFT88 accumulation in fibroblast cilia from affected individual UCL82 II.1 (Fig. 4); both indicate a retrograde IFT defect. If nearly normal amounts of IFT complexes A and B (apparently assembled into larger IFT trains; Fig. 6b) are being moved by fewer dynein motors, it could also explain why IFT velocity is reduced in the mutant flagella. In addition, since IFT dynein is reduced much more in the mutant flagella than in the cell body, the IFT dynein lacking Tctex2b is likely to be imported less efficiently into flagella than is the complete IFT dynein.

### TCTEX1D2 is a component of human IFT dynein

**Figure 7:**
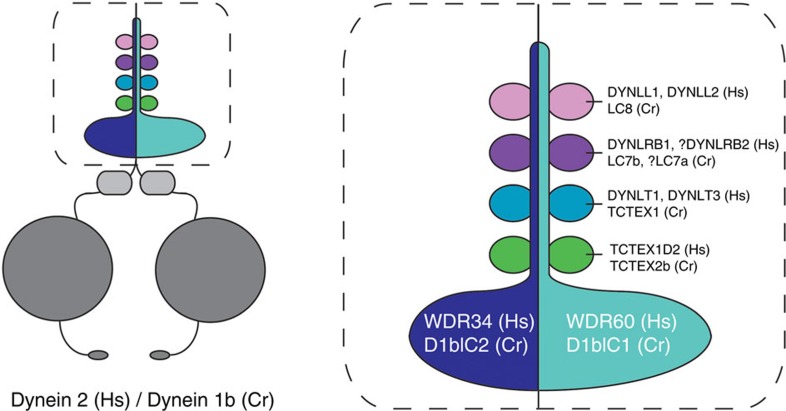
Proposed model of IFT dynein composition in *Homo sapiens* (Hs) and *C. reinhardtii* (Cr). Left, IFT dynein (dynein 2/1b) is composed of dynein heavy chains (DYNC2H1(Hs)/DHC1b(Cr); shown in dark grey), dynein light-intermediate chains (DYNC2LI1(Hs)/D1bLIC(Cr); shown in light grey) and different dynein intermediate and light chains (coloured, shown in detail on the right). Right, dynein intermediate chains (WDR34(Hs)/D1bIC2(Cr) and WDR60(Hs)/D1bIC1(Cr)) interact with different dynein light-chain subtypes, including TCTEX1D2(Hs)/TCTEX2b(Cr). Question marks indicate LC7a and DYNLRB2 as unconfirmed components suggested from our *Chlamydomonas* and human results.

To confirm that TCTEX1D2 is part of human IFT dynein, we performed affinity proteomics after expressing SF-TAP (Streptavidin/FLAG Tandem Affinity Purification)-tagged TCTEX1D2 in HEK293T cells; cells expressing SF-TAP-RAF1 served as a control. We were able to identify interactions of TCTEX1D2 with IFT dynein intermediate chains WDR34 and WDR60, and light chains DYNLT1, DYNLT3 and DYNLRB1 (Supplementary Table 7). No peptides from cytoplasmic dynein 1 intermediate chains were detected, ruling out possible contamination by that dynein. An interaction between TCTEX1D2 and WDR60 was verified after overexpression of Flag-tagged TCTEX1D2 and GFP-tagged WDR60 in HEK293T cells (Supplementary Fig. 8). Identification of TCTEX1D2, DYNLT1, DYNLT3 and DYNLRB1 as part of the human IFT dynein is consistent with our finding that the homologous Tctex2b, Tctex1 and LC7b are part of IFT dynein in *Chlamydomonas*, and provides independent evidence that IFT dynein contains several different light chains. A model of IFT dynein based on these findings is shown in Fig. 7.

## Discussion

Here we report that TCTEX1D2/Tctex2b is a light chain of IFT dynein in humans and *Chlamydomonas*, and show for the first time that it is essential for IFT dynein stability and normal retrograde (tip-to-base) IFT. In zebrafish, *tctex1d2* gene silencing suggests a role conserved across vertebrates. We show that biallelic loss-of-function alleles in this gene in humans cause JATD. In all affected individuals, we identified biallelic *TCTEX1D2* null alleles, which is in striking contrast to JATD and SRPS individuals carrying mutations in other IFT dynein genes such as *DYNC2H1* (refs 15, 16, 34, 38, 39, 39), *WDR34* (refs 12, 13), *WDR60* (ref. 14) or in genes encoding IFT-A^21^ and IFT-B^18^,^20^ components. These individuals usually carry at least one missense allele with inheritance restricted to two such hypomorphic alleles or one hypomorphic allele in combination with a null mutation^13^,^17^,^39^. Biallelic null alleles also cause early embryonic lethality in mouse IFT dynein knockouts^25^,^26^,^40^,^41^,^42^, and complete loss of IFT dynein subunits was thus to date considered incompatible with embryonic development beyond midgestation. Our data suggest that *TCTEX1D2* is an exception to this dogma, since all but one child carrying *TCTEX1D2* null mutations survived beyond infancy.

The high survival rate could reflect a milder thorax phenotype than in individuals with mutations in other IFT dynein genes such as *DYNC2H1*, *WDR34* and *WDR60* (refs 12, 13, 14, 15, 34). Similarly to individuals with mutations in the IFT dynein heavy-chain gene *DYNC2H1* (refs 15, 34), individuals with *TCTEX1D2* mutations did not show signs of extraskeletal disease; however, these phenotypes could still emerge with age. Two siblings (UCL4 II.1 and II.5) carrying homozygous *TCTEX1D2* mutations did not show overt signs of JATD, suggesting disease non-penetrance. Both are now young adults, and it is possible that early JATD radiological features (handlebar clavicles, trident acetabulum with spurs and cone-shaped epiphyses) were missed because a full X-ray exam was only performed in adulthood after we detected the unusual penetrance pattern. However, in contrast to their three affected siblings, they also do not exhibit polydactyly or shortened ribs. Although both are short statured with mildly short digits and lower limbs, more specific radiological signs of JATD/SRPS were not found.

Together, these clinical findings suggest a less essential role of TCTEX1D2 in ciliary transport mechanisms compared with other proteins mutated in JATD and SRPS. Cilia of human fibroblasts with *TCTEX1D2* mutations and *tctex1d2* zebrafish morphants lack gross structural defects, indicating a potentially non-essential role in vertebrate ciliogenesis. Similarly, flagella of the *Chlamydomonas tctex2b* null mutant are normal length at steady state, whereas null mutants of IFT dynein subunits DHC1b or D1bLIC have a severe short flagellar phenotype^43^,^44^,^45^.

Our studies in *Chlamydomonas* provide insight into the molecular mechanism underlying the milder phenotype. Loss of *Chlamydomonas* Tctex2b causes IFT dynein instability and a reduced amount of IFT dynein in flagella; however, surprisingly, the residual IFT dynein lacking Tctex2b retains enough functionality to maintain full-length flagella. Thus, it appears that a key role of Tctex2b is to stabilize IFT dynein. The lower level of IFT dynein is accompanied by reduced retrograde IFT velocity and frequency, causing an accumulation of IFT-particle proteins both in *Chlamydomonas* flagella and skin fibroblast cilia from affected individual UCL82 II.1. That *Chlamydomonas* cells tolerate some reduction of retrograde IFT is in agreement with observations that the *dhc1b* temperature-sensitive mutants *dhc1b-3* and *fla24* have reduced amounts of IFT dynein and reduced retrograde IFT at permissive temperature, yet still assemble normal length flagella^46^,^47^,^48^. In the case of the *tctex2b* mutant, flagella are formed more slowly than in wild-type cells, probably reflecting slower recycling of IFT components.

The slower flagellar assembly in the *Chlamydomonas tctex2b* mutant, if recapitulated in vertebrates, might explain our observation that *tctex1d2* morphant zebrafish embryos appeared to have short cilia at 24 h.p.f. but normal length cilia at 48 h.p.f. Interestingly, this finding, and the normal length cilia and flagella found in patient UCL82 II.1 fibroblasts and the *Chlamydomonas tctex2b* mutant, contrasts with a recent report that short interfering RNA-mediated depletion of *TCTEX1D2* in human telomerase-immortalized retinal pigment epithelial (hTERT-RPE1) cells resulted in longer cilia^49^. However, cilia length changes, ranging from shorter, normal length or longer than normal^12^,^18^,^34^, have been reported in cells from JATD patients with a number of gene defects. Furthermore, *WDR34* mutant patient fibroblasts have shortened cilia^12^, whereas *WDR34* short interfering RNA knockdown in hTERT-RPE1 results in longer cilia^49^. Vertebrate cilia length changes arising from retrograde IFT perturbations may thus be cell type specific or subject to experimental variability. These changes are not necessarily predictable, and therefore do not seem to provide a reliable measure, compared with the direct IFT measurements reported here that include immunofluorescence imaging of altered localization of IFT cargos and DIC imaging of IFT velocity. Although no difference in cilia length was observed in skin fibroblasts of JATD compared with control individuals in our study, similar to the *Chlamydomonas Tctex2b* mutant, any delay in cilia assembly could have severe consequences during development if full-length cilia are required for proper functioning of the hedgehog or other vertebrate ciliary signalling pathways soon after onset of cilia formation. Since retrograde IFT is critical for hedgehog signalling^11^,^50^, impaired retrograde IFT even in full-length cilia could be expected to affect skeletal development, as seen in the *TCTEX1D2*-mutated JATD individuals.

The *Chlamydomonas* Tctex2b mutant swims ∼30% slower than wild-type cells owing to defects in axonemal dyneins^35^. However, a ciliary motility defect is not the cause of JATD, because primary ciliary dyskinesia (PCD) patients with ciliary motility defects do not have JATD symptoms, and multiciliated cells bearing motile cilia are not present in the tissues affected in JATD nor in their progenitor cells^1^. JATD is caused by defects in primary cilia; primary cilia lack axonemal dyneins.

Proteomic analysis in *Chlamydomonas* and human cells confirmed Tctex2b/TCTEX1D2 as a component of IFT dynein along with intermediate chains D1bIC2/WDR34 and D1bIC1/WDR60, and a number of light chains. Immunoprecipitation of *Chlamydomonas* IFT dynein not only identified Tctex2b but also Tctex1 and LC7b as novel subunits of IFT dynein, indicating that these light chains together with LC8, D1bIC2 and D1bIC1 likely form a light-chain/intermediate chain complex within IFT dynein, which also may contain subunits not detected by our methods. Proteomics analysis of HEK293T proteins co-immunoprecipitated with SF-TAP-tagged TCTEX1D2 likewise identified DYNLT1 and DYNLT3 (human Tctex1), and additional light chains in the LC8 and roadblock families (DYNLL2=LC8-type 2 and DYNLRB1=DNLC2A). The light-chain pairs DYNLL1/DYNLL2 and DYNLRB1/DYNLRB2 have a high sequence identity of 93% and 77% respectively. This approach did not identify any peptides specific for DYNLL1 or DYNLRB2, but one peptide each was identified for DYNLL1/DYNLL2 and DYNLRB1/DYNLRB2, which could have originated from either of the two homologous proteins. Furthermore DYNLL1 was previously confirmed as a light chain of human IFT dynein^13^. Therefore, we propose that these subunits are components of an ancient, highly conserved intermediate chain/light-chain complex within IFT dynein (Fig. 7). As such, these light chains are strong candidates for harbouring novel mutations causing JATD.

Dynein complexes fall into two general types based on whether they contain single or multiple heavy-chain motor units^51^. Those with multiple motors all associate with a core group of accessory proteins including WD-repeat intermediate chains and light chains in the Dynll/LC8, Dynlt/Tctex1 and Dynlrb/LC7 classes (axonemal outer dynein arms and inner arm I1/f also include proteins related to TCTEX1D2/Tctex2, which form a distinct subfamily within the Dynlt/Tctex1 group^51^). Our demonstration that the IFT dynein includes a member of this subfamily suggests that the presence of TCTEX1D2 is also a conserved defining feature of these multimotor dyneins, and raises the possibility that canonical cytoplasmic dynein 1 might also associate with members of this light chain type under certain circumstances^52^.

The possible disease non-penetrance in family UCL4 is of clinical interest. Variable phenotypic severity is well documented for JATD/SRPS even within families. However, never before to the extent that individuals can appear clinically unaffected but carry biallelic loss-of-function variants that cause documented JATD/SRPS in other family members. Functional redundancy of TCTEX1D2 with other proteins could explain this, if, for example, other dynein light chains might in certain conditions be able to compensate for its loss. Mice carrying *Dynll1* mutations showed possible functional redundancy of IFT dynein light chains, whereby DYNLL2 may be able to function in place of DYNLL1 (ref. 53). We looked at single-nucleotide polymorphism haplotypes across dynein light-chain genes *DYNLL1*, *DYNLL2* and *TCTEX1* in individuals UCL4 II.1 and II.5 who carry the *TCTEX1D2* deletion but appear clinically unaffected. Both have different haplotypes across *TCTEX1* compared with their siblings II.8 and II.9 who display a classical JATD phenotype, but whether protective alleles could exist in compensatory genes is not clear (Supplementary Fig. 9).

Alternatively, TCTEX1D2 loss could be attenuated by individual-to-individual variation in the proteins with which TCTEX1D2 normally interacts, or in other proteins involved in IFT dynein stabilization or degradation. Modifier alleles have been debated as the underlying cause of the phenotypic variability in ciliary chondrodysplasias^13^,^23^,^34^ and other ciliopathies such as Bardet–Biedl, Joubert and Meckel syndromes; however, this has remained a challenge to prove in human subjects^54^,^55^. Variable penetrance in autosomal recessive conditions is still quite unusual, one example being a common *CFTR* variant that causes either disease or a normal phenotype depending on its genetic context^56^.

It may be relevant that partial suppression of mutations in genes encoding IFT-particle proteins has been observed in *Chlamydomonas* and *Tetrahymena*^57^,^58^,^59^; this occurs in association with stress, suggesting possible involvement of a chaperone in stabilizing the incomplete IFT particle. Given that a key role of Tctex2b is to stabilize IFT dynein, the presence or absence of another protein such as a chaperone that helps stabilize IFT dynein could determine whether or not a developmental defect is observed in the absence of Tctex2b. Interestingly, peptides derived from chaperones were abundant in our proteomic analysis of TAP-tagged TCTEX1D2 from HEK293T cells.

In summary, we have identified mutations associated with a complete loss of *TCTEX1D2* causing Jeune syndrome. *TCTEX1D2* mutations are a rare cause of disease affecting <5% of cases in this study (three affected families identified after screening of >300 individuals with JATD/SRPS). There are important implications for genetic counselling since individuals carrying *TCTEX1D2* mutations appear to have a higher survival rate than individuals affected by mutations in other IFT dynein components, and lack extraskeletal symptoms. In humans, fish and *Chlamydomonas*, *TCTEX1D2* mutations do not confer changes in gross ciliary structure, but do impair the highly conserved retrograde IFT machinery. Proteomic analysis provides evidence that TCTEX1D2/Tctex2b is a component of an intermediate chain/light-chain subcomplex within IFT dynein. In *Chlamydomonas*, the retrograde IFT defect is associated with instability of IFT dynein; motor stability may be the major role of Tctex2b since the residual IFT dynein missing Tctex2b retains some functionality. Our findings that IFT is impaired to a lesser extent in TCTEX1D2/Tctex2b null cells than in cells carrying null mutations in other IFT dynein components could explain, in part, the apparently incomplete penetrance underlying *TCTEX1D2* disease. This potentially has future clinical therapeutic implications, for example, if modulation of other components affecting the IFT dynein system could be harnessed to attenuate the effects of a lack of TCTEX1D2 function.

## Methods

### Patients

Inclusion criteria were the clinical diagnosis of JATD based on clinical and radiological findings including short ribs with small/narrow thorax and small ilia with acetabular spurs, handlebar clavicles and brachydactyly. All samples were obtained with approval of the UCL-ICH/Great Ormond Street Hospital Research Ethics Committee (08/H0713/82), South Yorkshire Research Ethics Committee (11/H1310/1) and collaborating institutions with informed consent.

### Human Sanger sequencing and PCR

Familial segregation in accordance with a recessive inheritance pattern was confirmed by Sanger sequencing of PCR products amplified from genomic DNA samples of all available family members (SourceBiosciences, Cambridge, UK). For family UCL4, fresh repeat samples were collected for segregation analysis, indicating no chance of sampling error. For RT–PCR, the Omniscript kit (Qiagen) was used to make cDNA from total RNA isolated from lymphocytes or fibroblasts of affected individuals and controls using Trizol–chloroform extraction. PCR and RT–PCR primers used are listed in Supplementary Table 1.

### Western blotting

Uncropped images of all western blots are included in Supplementary Fig. 10.

### Constructs used in mammalian cells

Mouse *WDR60* and human *TCTEX1D2* cDNA were obtained from the University of Queensland's SRC Microarray facility. A sequence-verified SF-TAP-*TCTEX1D2* construct (BC021177.2) containing a double Streptavidin II and a single FLAG tag was kindly provided by Nicholas Katsanis (Duke University, Durham, USA). *WDR60* was cloned into the GFP-N1 vector by PCR amplification, EcoRI/KpnI digestion and ligation. *TCTEX1D2* cloned into a modified pCR3 vector for expression of FLAG-tagged proteins was a kind gift from Dr Jörg Heierhorst (St Vincent's Institute of Medical Research, Melbourne). The human tGFP-tagged *WDR34* mammalian expression plasmid was obtained from OriGene Technologies, Inc. (TrueORF clone RG204288).

### Human cell culture

Human fibroblasts obtained by skin biopsy from study subjects and HEK293T cells (European Collection of Cell Cultures) were cultured under standard conditions at 37 °C and 5% CO_2_ in DMEM-F12 Glutamax medium (Life Technologies) with 10% fetal bovine serum (Life Technologies).

### Immunofluorescence in mammalian cells and zebrafish

Human fibroblasts were split onto glass coverslips and grown until confluent. For ciliogenesis experiments, cells were serum starved using cell medium without fetal bovine serum for 20 h. For immunofluorescence, cells were then fixed in 4% paraformaldehyde (PFA) for 10 min, washed five times with PBS, treated with 0.05% Triton X-100/PBS for 2 min, washed five times with PBS, blocked 1 h using 4% BSA in PBS and then incubated with the primary antibody. These were mouse monoclonal anti-acetylated tubulin 1:1,000 (IgG2b, clone 6-11-b1, Sigma), mouse monoclonal anti-pericentrin IgG1 1:200 (mAbcam 28144, Abcam) or rabbit polyclonal anti-IFT88 1:100 (13967-1-AP, Proteintech) overnight at 4 °C. Cells were then washed again five times with PBS and incubated with the appropriate secondary antibodies: goat anti mouseIgG1 Alexa Fluor 647, goat anti-mouse IgG2b Alexa Fluor 568 or goat anti-rabbit Alexa Fluor 488. Glass slides were then washed five times in PBS, incubated with 4,6-diamidino-2-phenylindole (Molecular Probes, Invitrogen) for 5 min to obtain nuclear stain, washed five times in PBS and then mounted in Vectashield (Vector Laboratories). For zebrafish immunofluorescence studies, embryos were fixed at 24 h.p.f. in 4% PFA overnight, washed five times in PBS containing 0.1% Triton X-100/PBS, incubated in 10% methanol for 30 min at −20 °C, washed five times in PBS containing 0.1% Triton X-100 and blocked with 5% BSA in PBS containing 0.1% Triton X-100 for 1 h. Embryos were then incubated with the primary antibody mouse monoclonal anti-acetylated tubulin 1:1,000 (IgG2b, clone 6-11-b1, Sigma) or mouse monoclonal anti-gamma tubulin 1:250 (IgG1, Sigma) overnight at 4 °C, washed five times in PBS containing 0.1% Triton X-100/PBS and then incubated with the appropriate secondary antibodies: goat anti-mouse IgG1 Alexa Fluor 647 1:1,000 or goat anti-mouse IgG2b Alexa Fluor 488 1:1,000 and 4,6-diamidino-2-phenylindole 1:25,000. All imaging was performed using a Zeiss LSM710 confocal microscope.

### Statistical analysis

Statistical analysis of cilia number and cilia length was performed using Student's *t*-test with statistical analysis performed using GraphPad Prism.

### *Chlamydomonas* cells and culture conditions

*Chlamydomonas reinhardtii* strain 137c (*nit1, nit2, mt*+) from the *Chlamydomonas* Resource Center (University of Minnesota, St Paul, MN) was used as wild type. A54-e18 (*nit1-1, ac17, sr1*, mt+), which is the parent of *pf16*-D2 (a double mutant of *pf16* and *tctex2b*), and ‘*pf16*-D2 Resc. w/PF16,' which is *pf16*-D2 rescued for the *PF16* gene^35^,^60^, were from Elizabeth Smith (Dartmouth College, NH). The *dic5-1* insertional mutant was generated by transforming g1 cells (*nit1, NIT2*, mt+)^61^ with the 1.7-kb chimeric aph7′′ gene cut by HindIII from the Hyg3 plasmid^62^, followed by backcrossing of a transformant to wild-type cells. The D1bIC2-HA strain was generated by transformation of *dic5-1* with a gene encoding D1bIC2 with a C-terminal 3 × HA tag. Cells were grown in TAP medium^63^ or M medium I^64^ altered to have 0.0022 M KH_2_PO_4_ and 0.00171 M K_2_HPO_4_; cultures were either aerated with 5% CO_2_ and 95% air or grown on 24-well plates.

### Creation of DNA constructs for *Chlamydomonas* transformation

The wild-type *DIC5* gene was amplified by PCR from genomic DNA using primer pairs FAP133-2 (5′-TGTCCCGCTGCAGAGCAATG-3′) and FAP133-3 (5′-ACCCCGCCTCCTTGTCCTTG-3′). The blunt ends of the PCR product were modified by A-tailing, that is, an extra A was added at the 3′ ends of the product strands by Taq polymerase. The modified PCR product was cloned into pGEM-T (Promega, Madison, MI). To insert an HA tag into the *DIC5* gene just before the stop codon, primer pair FAP133-15 (5′-GTAGAGTGGCAGTGCCGGC-3′) and FAP133-16 (5′-GCGTGAAGTTGCCGCGCA-3′) was used to amplify the *DIC5* gene and vector. The PCR product was then ligated to the 3 × HA fragment excised from plasmid p3 × HA^65^ by SmaI.

### *Chlamydomonas* immunoprecipitation and mass spectrometry

Flagella membrane-plus-matrix fractions from wild-type and D1bIC2-HA cells were incubated with the anti-HA affinity matrix (Roche Diagnostics GmbH) overnight. The beads were washed three times with HMEK buffer (30 mM HEPES, pH 7.4, 5 mM MgSO_4_, 0.5 mM EGTA and 25 mM KCl) plus 0.01% NP-40. Protease Inhibitor Cocktail for plant cell and tissue extracts (Sigma-Aldrich) was added to prevent protein degradation. SDS–PAGE sample loading buffer was used to elute the proteins from the matrix. Proteins in the bound fractions from wild-type and D1bIC2-HA samples were then separated by SDS–PAGE and stained with Silver Stain Plus (Bio-Rad). Gel regions of interest were excised and analysed by mass spectrometry at the Proteomics and Mass Spectrometry Facility, University of Massachusetts Medical School, and the Vermont Genetics Network Proteomics Facility, University of Vermont^66^,^67^.

### Flagellar regeneration and analysis of IFT

For DIC imaging of steady-state flagella and the subsequent analysis of IFT, live cells were immobilized in 1% agarose and observed using an inverted microscope (Ti U; Nikon) equipped with DIC optics^59^. To determine the kinetics of flagellar regeneration, cells were deflagellated by the pH-shock method^68^ and allowed to regrow flagella under the same conditions as before deflagellation. Aliquots of the cell suspensions were removed at various times and fixed with 1% glutaraldehyde. Images of the fixed cells were acquired with an AxioCam camera, AxioVision 3.1 software, and an Axioskop 2 plus microscope (Zeiss). Flagellar lengths were measured using ImageJ (http://rsb.info.nih.gov/ij/index.html). Images were processed using Adobe Photoshop (Adobe Systems Incorporated, San Jose, CA).

## Additional information

**How to cite this article:** Schmidts, M. *et al*. *TCTEX1D2* mutations underlie Jeune asphyxiating thoracic dystrophy with impaired retrograde intraflagellar transport. *Nat. Commun*. 6:7074 doi: 10.1038/ncomms8074 (2015).

## Supplementary Material

Supplementary Information Supplementary Figures 1-10, Supplementary Tables 1-7, Supplementary Methods and Supplementary References

## Figures and Tables

**Table 1:** Summary of clinical findings in individuals with *TCTEX1D2* mutations.
