# Corrigendum: *TCTEX1D2* mutations underlie Jeune asphyxiating thoracic dystrophy with impaired retrograde intraflagellar transport

**DOI:** 10.1038/ncomms11270

**Published:** 2016-03-29

**Authors:** Miriam Schmidts, Yuqing Hou, Claudio R. Cortés, Dorus A. Mans, Celine Huber, Karsten Boldt, Mitali Patel, Jeroen van Reeuwijk, Jean-Marc Plaza, Sylvia E. C. van Beersum, Zhi Min Yap, Stef J. F. Letteboer, S. Paige Taylor, Warren Herridge, Colin A. Johnson, Peter J. Scambler, Marius Ueffing, Hulya Kayserili, Deborah Krakow, Stephen M. King, UK10K, Saeed Al-Turki, Carl Anderson, Richard Anney, Dinu Antony, Jennifer Asimit, Mohammad Ayub, Jeff Barrett, Inês Barroso, Jamie Bentham, Shoumo Bhattacharya, Douglas Blackwood, Martin Bobrow, Elena Bochukova, Patrick Bolton, Chris Boustred, Gerome Breen, Marie-Jo Brion, Andrew Brown, Mattia Calissano, Keren Carss, Krishna Chatterjee, Lu Chen, Sebhattin Cirak, Peter Clapham, Gail Clement, Guy Coates, David Collier, Catherine Cosgrove, Tony Cox, Nick Craddock, Lucy Crooks, Sarah Curran, Allan Daly, Petr Danecek, George Davey Smith, Aaron Day-Williams, Ian Day, Richard Durbin, Sarah Edkins, Peter Ellis, David Evans, I. Sadaf Farooqi, Ghazaleh Fatemifar, David Fitzpatrick, Paul Flicek, Jamie Floyd, A. Reghan Foley, Chris Franklin, Marta Futema, Louise Gallagher, Tom Gaunt, Daniel Geschwind, Celia Greenwood, Detelina Grozeva, Xiaosen Guo, Hugh Gurling, Deborah Hart, Audrey Hendricks, Peter Holmans, Jie Huang, Steve E. Humphries, Matt Hurles, Pirro Hysi, David Jackson, Yalda Jamshidi, David Jewell, Joyce Chris, Jane Kaye, Thomas Keane, John Kemp, Karen Kennedy, Alastair Kent, Anja Kolb-Kokocinski, Genevieve Lachance, Cordelia Langford, Irene Lee, Rui Li, Yingrui Li, Liu Ryan, Jouko Lönnqvist, Margarida Lopes, Daniel G. MacArthur, Mangino Massimo, Jonathan Marchini, John Maslen, Shane McCarthy, Peter McGuffin, Andrew McIntosh, Andrew McKechanie, Andrew McQuillin, Yasin Memari, Sarah Metrustry, Josine Min, Alireza Moayyeri, James Morris, Dawn Muddyman, Francesco Muntoni, Kate Northstone, Michael O’Donovan, Stephen O’Rahilly, Alexandros Onoufriadis, Karim Oualkacha, Michael Owen, Aarno Palotie, Kalliope Panoutsopoulou, Victoria Parker, Jeremy Parr, Lavinia Paternoster, Tiina Paunio, Felicity Payne, John Perry, Olli Pietilainen, Vincent Plagnol, Michael A. Quail, Lydia Quaye, Lucy Raymond, Karola Rehnström, J. Brent Richards, Sue Ring, Graham R S Ritchie, David B. Savage, Nadia Schoenmakers, Robert K. Semple, Eva Serra, Hashem Shihab, So-Youn Shin, David Skuse, Kerrin Small, Carol Smee, Artigas María Soler, Nicole Soranzo, Lorraine Southam, Tim Spector, Beate St Pourcain, David St. Clair, Jim Stalker, Gabriela Surdulescu, Jaana Suvisaari, Ioanna Tachmazidou, Jing Tian, Nic Timpson, Martin Tobin, Ana Valdes, Margriet van Kogelenberg, Parthiban Vijayarangakannan, Louise Wain, Klaudia Walter, Jun Wang, Kirsten Ward, Ellie Wheeler, Ros Whittall, Hywel Williams, Kathy Williamson, Scott G. Wilson, Kim Wong, Tamieka Whyte, Xu ChangJiang, Eleftheria Zeggini, Feng Zhang, Hou-Feng Zheng, Philip L. Beales, Lihadh Al-Gazali, Carol Wicking, Valerie Cormier-Daire, Ronald Roepman, Hannah M. Mitchison, George B. Witman

Nature Communications 6: Article number:7074 ; DOI: 10.1038/ncomms8074 (2015); Published: 06 05 2015; Updated: 03 29 2016

The financial support for this article was not fully acknowledged. The Acknowledgements should have included the following:

PLB was supported by the National Institute for Health Research Biomedical Research Centre at Great Ormond Street Hospital for Children NHS Foundation Trust and University College London.

