# Supplementary material for: TCTEX1D2 mutations underlie Jeune asphyxiating thoracic dystrophy with impaired retrograde intraflagellar transport
Source: Nat Commun. 2015 Jun 5;6:7074. doi: 10.1038/ncomms8074 (PMC4468853; doi:10.1038/ncomms8074)
Supplement: Supplementary Information — Supplementary Figures 1-10, Supplementary Tables 1-7, Supplementary Methods and Supplementary References [file ncomms8074-s1.pdf]

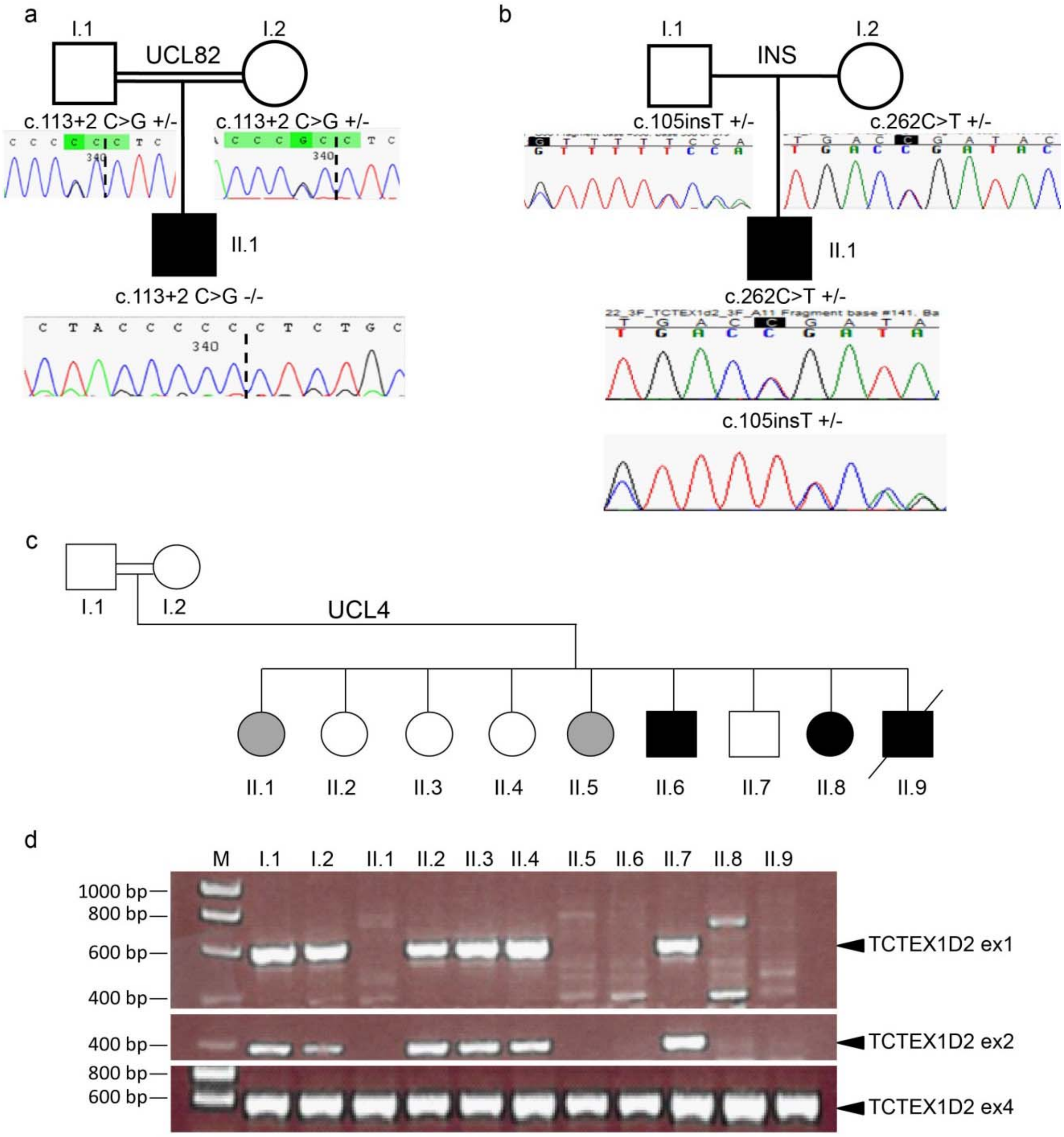

Supplementary Fig. 1. Segregation analysis in *TCTEX1D2* families. Pedigree and segregation analysis in (a) family UCL82 and (b) INS, both consistent with autosomal recessive inheritance. (c) family UCL4 and (d) genomic PCRs in UCL4 of *TCTEX1D2* exon 1 and exon 2 (affected by the deletion) plus exon 4 (not affected by the deletion). Children carrying the homozygous exon 1-2 *TCTEX1D2* deletion are marked in black (diagnosed with JATD) or grey (two siblings who were not diagnosed with JATD. The strikethrough indicates death at 2 months of age, double line indicates consanguineous marriage. See also main article Figure 1. M, DNA ladder marker.

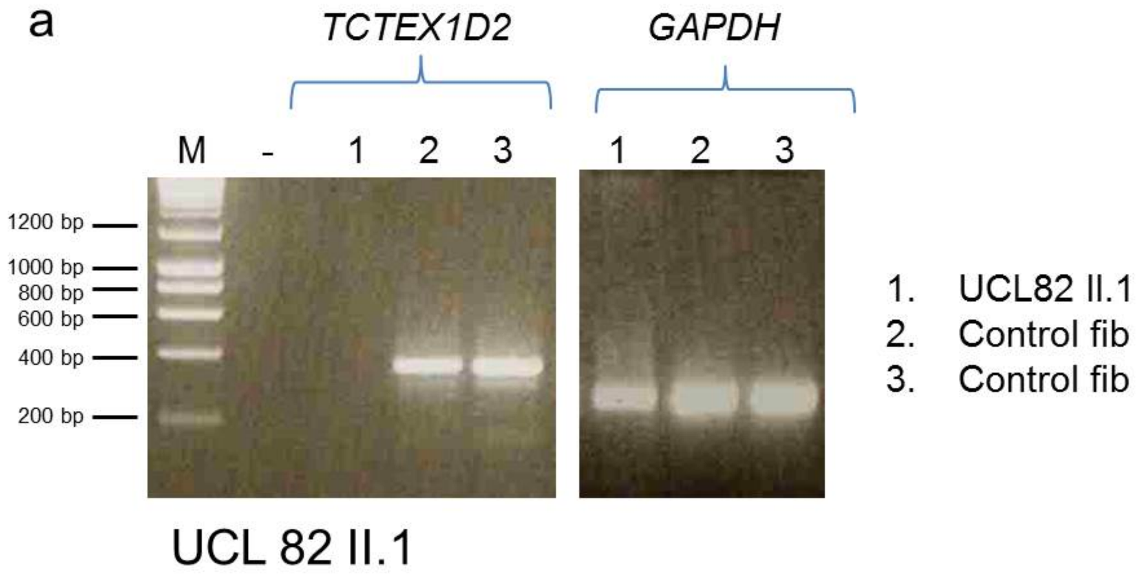

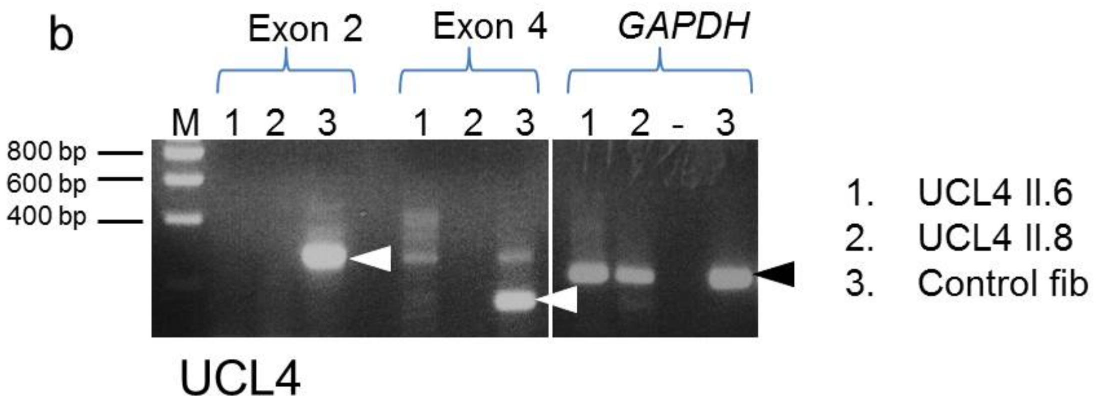

Supplementary Fig. 2. RT-PCR confirms absence of TCTEX1D2 transcript in family UCL82 and UCL4. (a) Reverse transcription (RT) PCR performed on RNA isolated from fibroblasts of individual UCL82 II.1 and a controls confirms the absence of transcript as a result of the splice defect c.113+2C>G. *GAPDH* is shown as positive control. The *TCTEX1D2* forward primer is in exon 1 and reverse primer in exon 5 (see online methods). (b) The effect on the *TCTEX1D2* transcript of the deletion in family UCL4 was assessed by RT-PCR performed on RNA isolated from blood lymphocytes of UCL4 II.6 and II.8. *TCTEX1D2* exon 2 and exon 4 did not amplify in the two affected individuals compared to the control. GAPDH was included as a positive control. M, DNA ladder marker.

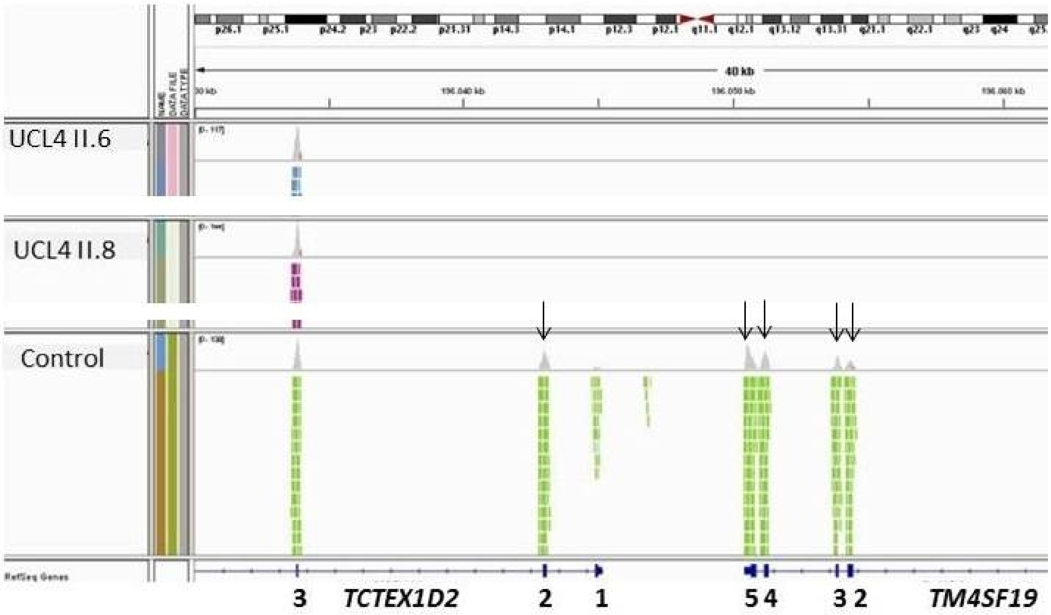

Supplementary Fig. 3. IGV screenshot visualising the deletion in family UCL4. Visualisation using Integrated Genome Viewer of the *TCTEX1D2* deletion encompassing exon 1-2 and exon 2-5 of the adjacent gene *TM4SF19*, exons numbered below. Input BAM files of individuals UCL4 II.6 and II.8 (upper and middle panel) and a control (lower panel) were generated from whole exome sequencing (UK10K project). Note that exon 1 of *TM4SF19* was found to be generally absent from all UK10K exomes, probably due to capture failure by the Agilent V2 kit.

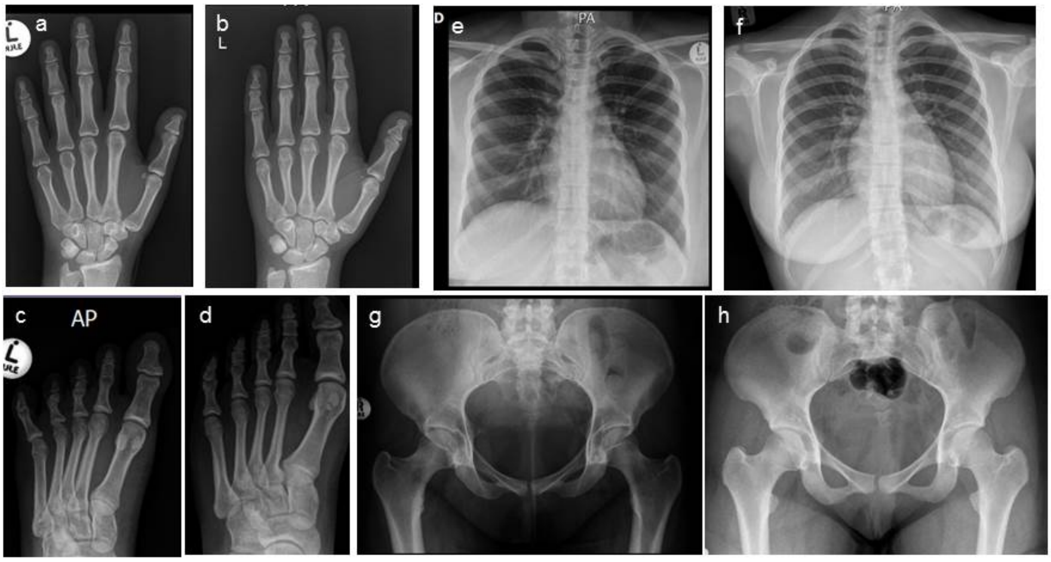

Supplementary Fig. 4. X-rays of individuals UCL 4 II.1 and II.5. Mild brachydactyly (a-d) and clinodactyly (c,d) but no rib shortening or handlebar clavicles (e,f) and no signs of trident acetabulum with spurs (g,h).

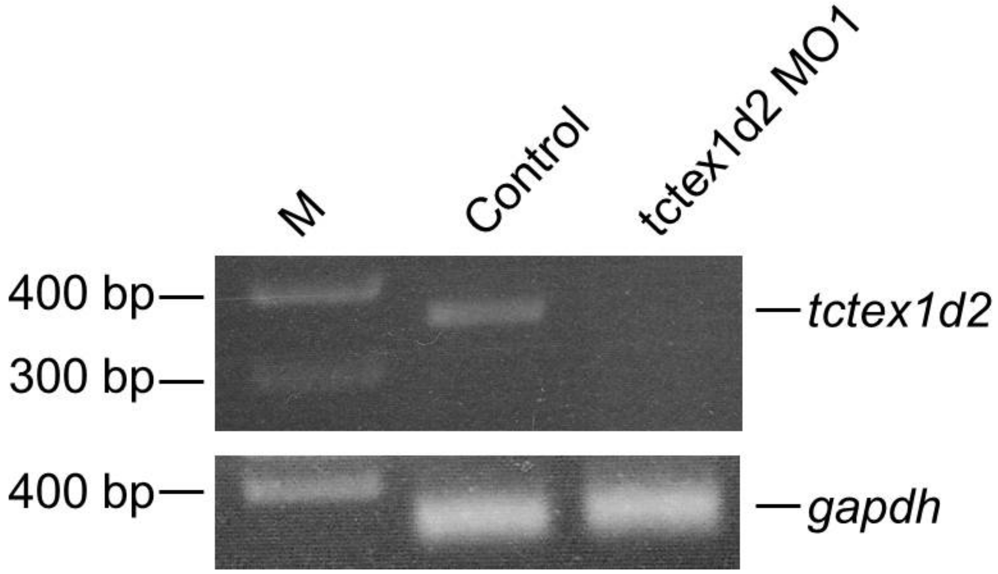

Supplementary Fig. 5. RT-PCR confirms loss of *tctex1d2* expression after morpholino injection. Using a forward primer in exon 1 and reverse primer in exon 5, no transcript could be amplified by RT-PCR in 48 hpf *tctex1d2* morphants compared to control morpholino-injected zebrafish embryos, indicating successful knockdown of the transcript (see online methods for details). M, DNA ladder marker.

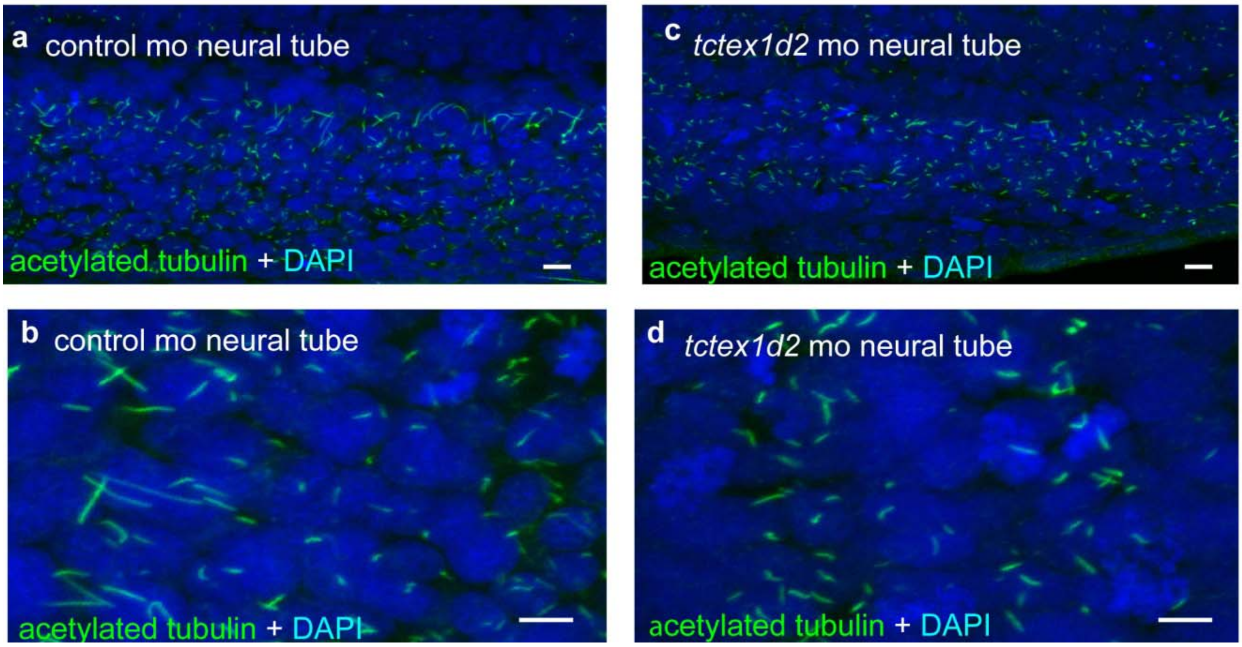

Supplementary Fig. 6. Ciliogenesis appears undisturbed in *tctex1d2* morphant neural tube. The appearance of neural tube cilia in control-morpholino (a, b) and *tctex1d2*-morpholino (c, d) injected zebrafish embryos was similar at 24hpf using whole mount confocal microscopy after staining with anti-acetylated tubulin antibody and DAPI (see online methods for details). Scale bar 10 µm.

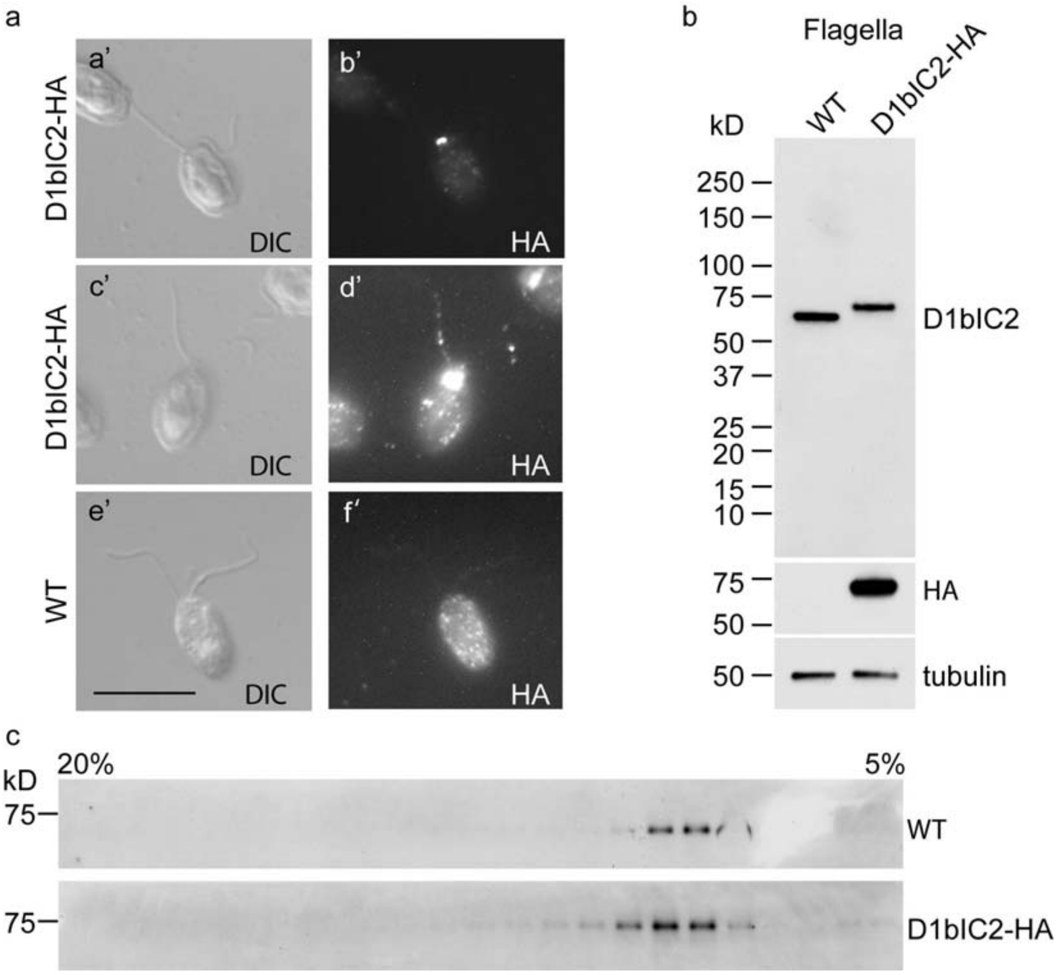

Supplementary Fig. 7. HA-tagged D1bIC2 in the D1bIC2-HA strain behaves the same as D1bIC2 in wild-type (WT) cells. (a) HA-tagged D1bIC2 localizes mainly in the basal body region with a few puncta along the flagella. D1bIC2-HA cells (a' to d') and control wild-type cells (e', f') were stained with anti-HA antibody and imaged by epifluorescence (b', d', f') and DIC (a', c', and e') microscopy. Image b' was taken by focusing on the basal body region. Image d' was taken by focusing on the flagella with image brightness increased to show labelling of the flagella; identical processing was used for images d', f'. The D1bIC2-HA labelling in D1bIC2-HA cells is identical to that reported for D1bIC2 in wild-type cells (see Fig. 2 in [1]); no basal body or flagellar signal was detected in wild-type cells, which lack HA-tagged D1bIC2. Note also that the D1bIC2-HA cells assemble full-length flagella. Scale bar 10 µm. (b) HA-tagged D1bIC2 is imported into flagella. Western blots of flagella from wild-type and the strain expressing HA-tagged D1bIC2 were probed with anti-D1bIC2 antibody and anti-HA antibody. D1bIC2 and D1bIC2-HA are present at similar levels in flagella of the wild-type and D1bIC2-HA strains, respectively; D1bIC2-HA migrates above D1bIC2 due to its 3xHA tag (4.4 kDa). No wild-type D1bIC2 was detected in the D1bIC2-HA strain. The same samples were diluted and probed for tubulin as loading control. (c) Complexes containing wild-type D1bIC2 and D1bIC2-HA sediment identically in sucrose gradients. Flagellar membrane-plus-matrix preparations from wild-type and the strain expressing HA-tagged D1bIC2 were fractionated on a 5-20% sucrose gradient. The fractions were collected and analyzed in western blots probed with anti-D1bIC2 antibody (WT sample) or anti-HA antibody (D1bIC2-HA strain). Both proteins peak identically at ~10S; wild-type D1bIC2 previously was shown to peak at ~10S in sucrose gradients[1].

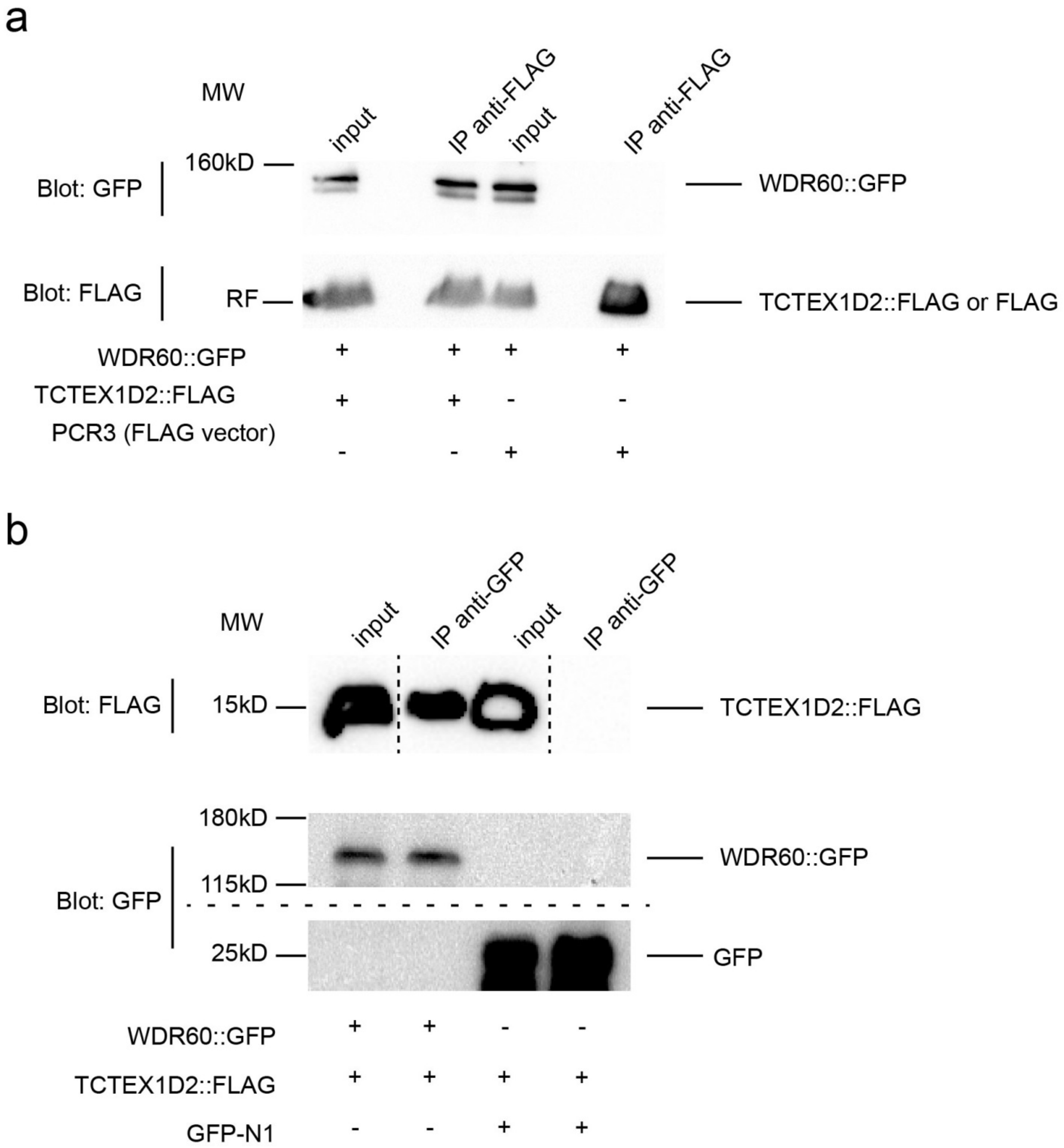

Supplementary Fig. 8. TCTEX1D2 interacts with WDR60 *in vitro*. (a) HEK293T cells were transfected with either empty FLAG vector (PCR3) and WDR60::GFP or TCTEX1D2::FLAG and WDR60::GFP. Anti-FLAG IP was performed using a standard protocol, showing that WDR60::GFP co-precipitates with TCTEX1D2::FLAG only. Relative levels of overexpressed proteins were verified in the corresponding input samples. (b) The reverse immunoprecipitation (anti-GFP IP) confirms the TCTEX1D2-WDR60 interaction; TCTEX1D2::FLAG co-precipitates with WDR60::GFP, but does not co-precipitate with the GFP tag (GFP-N1 vector). Vertical dashed lines indicate cropping for removal of empty lanes in FLAG blot. Horizontal dashed lines indicate discontinuity of GFP blot due to marked difference in sizes of WDR60::GFP and GFP tag. RF, running front. MW, molecular weight marker. kD, kilodaltons.

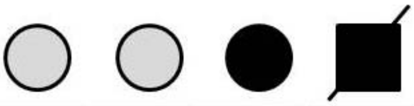

SNP	position	II.1	II.5	II.8	II.9
		31_C7	31_C3	31_C8	31_C9
DYNLL2					
SNP_A-1865694	56600056	AB	AB	AB	AB
SNP_A-1865703	56615425	AB	AB	BB	AB
SNP_A-2231510	56618151	AB	AB	AA	AB
**DYNLT1 (TCTEX1)**					
**SNP_A-4235563**	**158913155**	**AA**	**AA**	**AA**	**AB**
**SNP_A-2168029**	**159055851**	**AA**	**AB**	**AB**	**AB**
**SNP_A-2218526**	**159066859**	**AA**	**AB**	**AA**	**AA**
**SNP_A-2234501**	**159066919**	**BB**	**AB**	**AB**	**AB**
DYNLL1					
SNP_A-1857328	120935570	AB	BB	AB	AB

Supplementary Fig. 9. Haplotype analysis in family UCL4 for IFT dynein light chain genes other than *TCTEX1D2*.

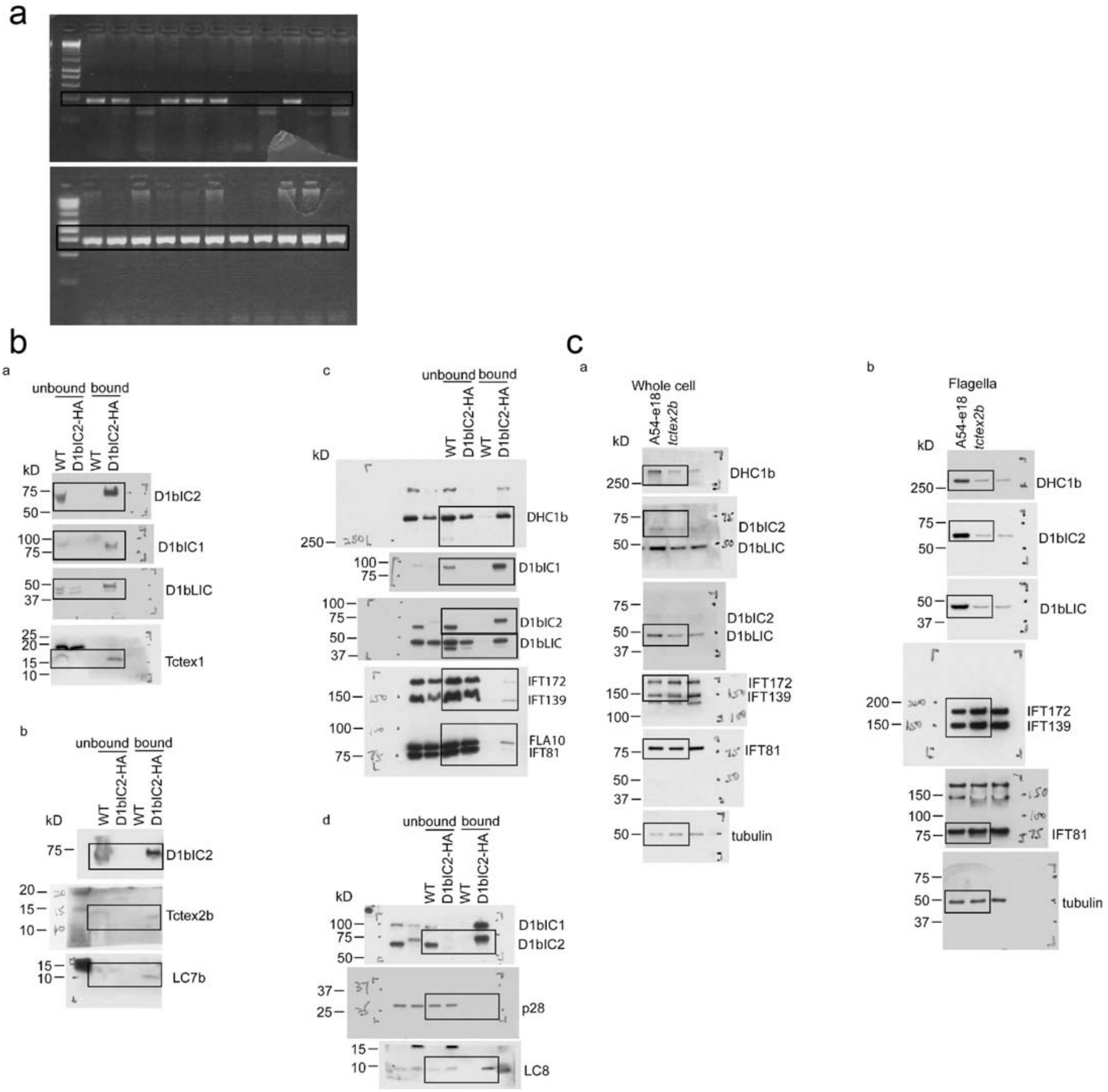

Supplementary Fig. 10. Representative original uncropped images of key agarose gels and western blots. (a) Images of the selected portions shown in Figure 1a are highlighted in the black boxes. (b) Images of the selected portions shown in Figure 5b-e are highlighted in the black boxes. (c) Images of the selected portions shown in Figure 6d,e are highlighted in the black boxes (two different exposures of the same blot were used for D1bIC2 and D1bLIC in 6d, as shown).

Gene	Amplicon	Size (bp)	Primers (5'-3')
Hs. TCTEX1D2	Exon 1 genomic	594	CGCGTCTATTTCCTCCGACT<br>AAACACTGAGGAAGCGGGTG
Hs. TCTEX1D2	Exon 2 genomic	397	GTGCAGGTCCAGGTTGAAGA<br>GATGGAGAGGGGGCAAATGG
Hs. TCTEX1D2	Exon 3 genomic	311	GATCTGCCTTCACGTCCTCC<br>AGGCACTCTTACTGAATTGTCCA
Hs. TCTEX1D2	Exon 4 genomic	599	TTTCTGTGCCTTCCTCGTCC<br>TCCTTGCCTATAAGCAGCACT
Hs. TCTEX1D2	Exon 5 genomic	200	TGGAAAGATCTGAGATGGTCTT<br>AGACATGACCATGAAGAAATCTGA
Hs. GAPDH	RT-PCR control gene	231	GAGAAGGCTGGGGCTCATTT<br>AGTGATGGCATGGACTGTGG
Hs. TCTEX1D2	RT-PCR UCL82 splice	326	TGAGGCTGAGAAGAACGCAG<br>TGCGTTGTAGCAGCATTTGG
Hs. TCTEX1D2	RT-PCR UCL4 exon 2	257	CATCGGAGTGTCCTTCTCGG<br>TGGTGGTGCAAGTAGTGATTG
Hs. TCTEX1D2	RT-PCR UCL4 exon 4	120	ACCGATACAAAATGGTGGTGC<br>GCGTTGTAGCAGCATTTGGC
Dr. tctex1d2	RT-PCR Morpholino 1	370	AAGCGTGATATTTCACTCATGGAT<br>CAGCAAAAGCAGCAGCTACAC
Dr. gapdh	RT-PCR control gene	300	TTAAGGCAGAAGGCGGCAAA<br>AAGGAGCCAGGCAGTTGGTG

Supplementary Table 1. PCR and RT-PCR primers used for human (Hs.) and zebrafish (Dr.) genetic analysis.

Linkage analysis II.1 and II.5 coded as unknown	Genomic coordinates	Homozygous interval in Mb	LOD score	Variants shared in II.6 and II.8 within the homozygous interval from exome sequencing
Homozygous interval 1	Chr1: 30672845-42928730	12.255885	2.9056	-
Homozygous interval 2	Chr1: 232069057-234510262	2.441	2.9056	-
Homozygous interval 3	Chr3: 189033565-197102197	8.800193	2.9056	*TCTEX1D2* homoz deletion exon 1 + 2
Homozygous interval 4	Chr11: 13620172-17897324	4.277152	2.9056	-
Homozygous interval 5	Chr14: 87124570-90575080	3.45051	2.8819	-
Homozygous interval 5	Chr.17: 59927307-64779430	4.852123	2.9056	*TBC1D3P2, ENSG00000188755; pseudogene, no protein product,* non-canonical splice (c.282G>T, p. L94L)

Supplementary Table 2. Linkage analysis coded UCL II.1 and II.5 as unknown.

Linkage analysis II.1 and II.5 coded affected, penetrance 60%	Genomic coordinates	Homozygous interval in Mb	LOD score	Variants shared in II.6 and II.8 within the homozygous interval from exome sequencing
Homozygous interval 1	Chr3: 182883408-186915096	4.031688	3.8919	-
Homozygous interval 2	Chr3: 188452557-197102197	9.381201	3.8919	*TCTEX1D2* homoz deletion exon 1 + 2
Homozygous interval 3	Chr X: 13559816-22835253	9.275437	1.5227	-

Supplementary Table 3. Linkage analysis coding UCL II.1 and II.5 as affected assuming 60% penetrance.

Linkage analysis II.1 and II.5 coded as unaffected	Genomic coordinates	Homozygous interval in Mb	LOD score	Variants shared in II.6 and II.8 within the homozygous interval from exome sequencing
Homozygous interval 1	Chr.11: 13576748-17853900	4.277152	3.1554	-
Homozygous interval 2	Chr.14: 86242481 - 90168964	3.865594	3.1343	-
Homozygous interval 3	Chr.17: 57804047 - 62253083	4.927803	3.1554	*TBC1D3P2, ENSG00000188755; pseudogene, no protein product,* non-canonical splice (c.282G>T, p. L94L)

Supplementary Table 4. Linkage analysis coding UCL II.1 and II.5 as unaffected.

Linkage analysis II.1 and II.5 coded affected	Genomic coordinates	Homozygous interval in Mb	LOD score	Variants shared in II.6 and II.8 within the homozygous interval from exome sequencing
Homozygous interval 1	Chr.3 188452557-197102197	9.381201	4.1097	*TCTEX1D2* homoz deletion exon 1 + 2

Supplementary Table 5. Linkage analysis coding UCL II.1 and II.5 as affected.

Protein	Antibody	Type	Working dilution for blots	Reference
Chlamydomonas DHC1b	α-DHC1b	P	1:1000	Pazour et al., 1999[2]
Chlamydomonas D1bLIC	α-D1bLIC	P	1:2000	Hou et al., 2004[3]
Chlamydomonas FAP133	CT248	P	1:2000	Rompolas et al., 2007[1]
Chlamydomonas FAP163	CT295	P	1:10	Patel-King et al., 2013[4]
Chlamydomonas LC8	R4058	P	1:1000	King and Patel-King, 1995[5]
Chlamydomonas Tctex1	R5205	P	1:500	King et al., 1996[6]
Chlamydomonas Tctex2b	CT117	P	1:200	Dibella et al., 2004a[7]
Chlamydomonas LC7b	CT116	P	1:1000	Dibella et al., 2004b[8]
Chlamydomonas p28	α-p28	P	1:20,000	LeDizet and Piperno, 1995[9]
Chlamydomonas FLA10	α-FLA10	P	1:5000	Cole et al., 1998[10]
Chlamydomonas IFT139	139.1	M	1:100	Cole et al., 1998[10]
Chlamydomonas IFT172	172.1	M	1:100	Cole et al., 1998[10]
Chlamydomonas IFT81	81.1	M	1:500	Cole et al., 1998[10]
HA	3F10	R	1:2000;	Roche Diagnostics Corporation (Indianapolis, IN)

			1:400[IF]	
Chlamydomonas α-tubulin	B5-1-2	M	1:2000	Sigma-Aldrich, Inc. (St. Louis, MO)

Supplementary Table 6. Antibodies used for *Chlamydomonas* protein biochemistry. M: mouse monoclonal; P: rabbit polyclonal; R: rat monoclonal.

Proteins identified by mass spectrometry from tandem affinity proteomics experiments in HEK293T cells			TCTEX1D2_N-TAP_Exp1		TCTEX1D2_N-TAP_Exp2	
EntrezGene Symbol	EntrezGeneFullName	SwissProt_2013_12_ Accession	**unique peptides**	**sequence coverage**	**unique peptides**	**sequence coverage**
ABHD17B	abhydrolase domain containing 17B	Q5VST6	3	0.149		
ACACA	acetyl-CoA carboxylase alpha	Q13085	4	0.015		
AKAP8L	A kinase (PRKA) anchor protein 8-like	Q9ULX6	4	0.065	4	0.070
ATP2A2	ATPase, Ca++ transporting, cardiac muscle, slow twitch 2	P16615	6	0.071	4	0.060
ATP5A1	ATP synthase, H+ transporting, mitochondrial F1 complex, alpha subunit 1, cardiac muscle	P25705	7	0.163	5	0.108
AZGP1	alpha-2-glycoprotein 1, zinc-binding	P25311			2	0.064
BAG5	BCL2-associated athanogene 5	Q9UL15	3	0.087		
BLVRA	biliverdin reductase A	P53004	10	0.358	6	0.226
BSG	basigin (Ok blood group)	P35613	5	0.184	2	0.073
C1QBP	complement component 1, q subcomponent binding protein	Q07021	4	0.262	3	0.191
C2orf47	chromosome 2 open reading frame 47	Q8WWC4	2	0.062	2	0.079
CAMK2D	calcium/calmodulin-dependent protein kinase II delta	Q13557	5	0.134		
CANX	calnexin	P27824	10	0.194		
CDC42EP1	CDC42 effector protein (Rho GTPase binding) 1	Q00587	2	0.049		
CDIPT	CDP-diacylglycerol--inositol 3-phosphatidyltransferase	O14735	2	0.099		
CHCHD4	coiled-coil-helix-coiled-coil-helix domain containing 4	Q8N4Q1	3	0.359	2	0.232
COL1A1	collagen, type I, alpha 1	P02452			10	0.101
COL1A2	collagen, type I, alpha 2	P08123			6	0.047
COX15	cytochrome c oxidase assembly homolog 15 (yeast)	Q7KZN9	2	0.039		
CPT1A	carnitine palmitoyltransferase 1A (liver)	P50416	2	0.031		
CXorf57	chromosome X open reading frame 57	Q6NSI4	2	0.042		
DBT	dihydrolipoamide branched chain transacylase E2	P11182	2	0.033	4	0.087
DCD	dermcidin	P81605	3	0.264	2	0.200
DDB1	damage-specific DNA binding protein 1, 127kDa	Q16531	4	0.030		
DHRS7B	dehydrogenase/reductase (SDR family) member 7B	Q6IAN0	2	0.074		
DNAJA3	DnaJ (Hsp40) homolog, subfamily A, member 3	Q96EY1	4	0.106		
DNAJB1	DnaJ (Hsp40) homolog, subfamily B, member 1	P25685	2	0.062		
DNAJB11	DnaJ (Hsp40) homolog, subfamily B, member 11	Q9UBS4	3	0.075		
DNAJB12	DnaJ (Hsp40) homolog, subfamily B, member 12	Q9NXW2	2	0.053		
DNAJB6	DnaJ (Hsp40) homolog, subfamily B, member 6	O75190	11	0.350	7	0.248
DNAJC7	DnaJ (Hsp40) homolog, subfamily C, member 7	Q99615	40	0.682	38	0.664
DPM1	dolichyl-phosphate mannosyltransferase polypeptide 1, catalytic subunit	O60762	2	0.112		
DYNLL2	dynein, light chain, LC8-type 2	Q96FJ2	2	0.202		
DYNLRB1	dynein, light chain, roadblock-type 1	Q9NP97	2	0.292	2	0.292

DYNLT1	dynein, light chain, Tctex-type 1	P63172	8	0.965	7	0.770
DYNLT3	dynein, light chain, Tctex-type 3	P51808	3	0.440	4	0.733
EEF2	eukaryotic translation elongation factor 2	P13639	4	0.044		
EIF4G1	eukaryotic translation initiation factor 4 gamma, 1	Q04637	25	0.183	6	0.041
FAF2	Fas associated factor family member 2	Q96CS3	3	0.076		
FAM210A	family with sequence similarity 210, member A	Q96ND0	2	0.081		
FANCI	Fanconi anemia, complementation group I	Q9NVI1	5	0.044		
FAR1	fatty acyl CoA reductase 1	Q8WVX9	3	0.072		
FKBP8	FK506 binding protein 8, 38kDa	Q14318	2	0.056		
FLG	filaggrin	P20930	2	0.002		
GAPDH	glyceraldehyde-3-phosphate dehydrogenase	P04406	3	0.128	5	0.179
GLUD1	glutamate dehydrogenase 1	P00367,P49448	2	0.034		
HAX1	HCLS1 associated protein X-1	O00165	11	0.559	7	0.366
HLA-A	major histocompatibility complex, class I, A	P01892,P10316	2	0.079		
HLA-B	major histocompatibility complex, class I, B	P30480	6	0.213		
HNRNPH1	heterogeneous nuclear ribonucleoprotein H1 (H)	P31943	3	0.098		
HSD17B12	hydroxysteroid (17-beta) dehydrogenase 12	Q53GQ0	6	0.215	2	0.077
HSPA1L	heat shock 70kDa protein 1-like	P34931	2	0.321		
HSPA4	heat shock 70kDa protein 4	P34932	7	0.099		
HSPA5	heat shock 70kDa protein 5 (glucose-regulated protein, 78kDa)	P11021	10	0.223	3	0.127
HSPA9	heat shock 70kDa protein 9 (mortalin)	P38646	15	0.283	3	0.059
HSPB1	heat shock 27kDa protein 1	P04792	6	0.420	3	0.137
HSPH1	heat shock 105kDa/110kDa protein 1	Q92598	5	0.087		
HUWE1	HECT, UBA and WWE domain containing 1, E3 ubiquitin protein ligase	Q7Z6Z7	19	0.048	4	0.014
KRT6C	keratin 6C	P48668			2	0.509
LAMP2	lysosomal-associated membrane protein 2	P13473	2	0.049		
LCN1	lipocalin 1	P31025			2	0.125
LDHB	lactate dehydrogenase B	P07195	3	0.117		
MAD2L1	MAD2 mitotic arrest deficient-like 1 (yeast)	Q13257	6	0.278	3	0.205
MAGED1	melanoma antigen family D, 1	Q9Y5V3	3	0.039		
MAGED2	melanoma antigen family D, 2	Q9UNF1	3	0.051		
MLF2	myeloid leukemia factor 2	Q15773	3	0.161	2	0.085
MSH6	mutS homolog 6	P52701	2	0.016		
MTR	5-methyltetrahydrofolate-homocysteine methyltransferase	Q99707	2	0.015		
NDUFA4	NADH dehydrogenase (ubiquinone) 1 alpha subcomplex, 4, 9kDa	O00483	3	0.370	3	0.346
NME1-NME2	NME1-NME2 readthrough	P22392	6	0.467	3	0.191
NME2	NME/NM23 nucleoside diphosphate kinase 2	P22392	6	0.467	3	0.191
NME3	NME/NM23 nucleoside diphosphate kinase 3	Q13232	2	0.130		

PCNA	proliferating cell nuclear antigen	P12004	4	0.149		
PGAM5	phosphoglycerate mutase family member 5	Q96HS1	4	0.131		
PIP	prolactin-induced protein	P12273			2	0.185
PRKDC	protein kinase, DNA-activated, catalytic polypeptide	P78527	7	0.017		
PTPLAD1	protein tyrosine phosphatase-like A domain containing 1	Q9P035	4	0.155		
RBBP7	retinoblastoma binding protein 7	Q16576	2	0.056		
RPL23	ribosomal protein L23	P62829	3	0.257	3	0.271
RPL38	ribosomal protein L38	P63173	3	0.329		
RPS12	ribosomal protein S12	P25398	3	0.205		
RPS16	ribosomal protein S16	P62249	2	0.130		
RPS2	ribosomal protein S2	P15880	2	0.082		
RPS27	ribosomal protein S27	P42677	2	0.226		
RPS3	ribosomal protein S3	P23396	7	0.280		
RPS6	ribosomal protein S6	P62753	2	0.072		
S100A9	S100 calcium binding protein A9	P06702	4	0.377		
SDF4	stromal cell derived factor 4	Q9BRK5	5	0.160		
SEC61A1	Sec61 alpha 1 subunit (S. cerevisiae)	P61619	2	0.046		
SERPINB12	serpin peptidase inhibitor, clade B (ovalbumin), member 12	Q96P63	2	0.044	2	0.044
SF3B3	splicing factor 3b, subunit 3, 130kDa	Q15393	5	0.041		
SLC16A1	solute carrier family 16 (monocarboxylate transporter), member 1	P53985	2	0.064		
SLC25A11	solute carrier family 25 (mitochondrial carrier; oxoglutarate carrier), member 11	Q02978	6	0.207	5	0.182
SLC25A13	solute carrier family 25 (aspartate/glutamate carrier), member 13	Q9UJS0	8	0.123	3	0.043
SLC25A22	solute carrier family 25 (mitochondrial carrier: glutamate), member 22	Q9H936	8	0.241	6	0.189
SLC25A6	solute carrier family 25 (mitochondrial carrier; adenine nucleotide translocator), member 6	P12236	4	0.530	4	0.493
SLC3A2	solute carrier family 3 (amino acid transporter heavy chain), member 2	P08195	6	0.129	3	0.068
SLC7A5	solute carrier family 7 (amino acid transporter light chain, L system), member 5	Q01650	2	0.049		
SPRR2E	small proline-rich protein 2E	P22531,P22532,P35325,P35326,Q9BYE4	2	0.306		
SSR1	signal sequence receptor, alpha	P43307	2	0.080	3	0.119
SSR4	signal sequence receptor, delta	P51571	4	0.306	3	0.243
STX17	syntaxin 17	P56962	2	0.096		
TARS	threonyl-tRNA synthetase	P26639	2	0.025		
TCTEX1D2	Tctex1 domain containing 2	Q8WW35	11	0.627	11	0.627
TECR	trans-2,3-enoyl-CoA reductase	Q9NZ01	2	0.068		
TIMM21	translocase of inner mitochondrial membrane 21 homolog (yeast)	Q9BVV7	3	0.137		
TIMM50	translocase of inner mitochondrial membrane 50 homolog (S. cerevisiae)	Q3ZCQ8	5	0.187	2	0.076

TIPRL	TOR signaling pathway regulator	O75663	4	0.154		
TUBA1C	tubulin, alpha 1c	Q9BQE3	3	0.595	2	0.563
TUBB2A	tubulin, beta 2A class IIa	Q13885	4	0.569	4	0.557
TUBB4A	tubulin, beta 4A class IVa	P04350	3	0.649	2	0.631
TUBB6	tubulin, beta 6 class V	Q9BUF5	5	0.437	2	0.336
TXN	thioredoxin	P10599	2	0.229		
UBA52	ubiquitin A-52 residue ribosomal protein fusion product 1	P62987	5	0.367		
USMG5	up-regulated during skeletal muscle growth 5 homolog (mouse)	Q96IX5	2	0.431	2	0.431
WDR34	WD repeat domain 34	Q96EX3	8	0.192	5	0.140
WDR60	WD repeat domain 60	Q8WVS4	22	0.260	8	0.108
XPO1	exportin 1	O14980	8	0.110		
XPOT	exportin, tRNA	O43592	2	0.018		
YTHDF2	YTH domain family, member 2	Q9Y5A9	3	0.060		

Supplementary Table 7. SF-TAP of N-terminally over-expressed *TCTEX1D2* in HEK293T cells showing the number of uniquely identified peptides and sequence coverage for each protein detected by mass spectrometry. DYNLL1 and DYNLRB2 are not listed as only one peptide was identified which could not be specifically attributed. Proteins identified in the SF-TAP analysis of the unrelated RAF1 control protein are not shown. IFT dynein components are marked in red.

Supplementary methods

Whole Exome Sequencing (WES)

Exomes (69) generated at UCL were prepared using the Agilent V2 (UK10K project) or Truseq whole exome kit with sequencing of enriched libraries performed as 75 base paired-end reads (Illumina HiSeq)[11-13]. Exomes (154) generated at UCLA were sequenced at the University of Washington Center for Genome Sciences, captured using the NimbleGen SeqCap EZ Exome Library v2.0 probe library and sequenced on the Illumina GAIIx platform with 50 bp reads. For both sets of data, passed reads were mapped to human genome reference hg19 using BWA v0.5.9r16[14] with realignment around known indels from the 1000 Genomes Pilot study and recalibration of base quality scores performed using GATK 1.1.5[15]. Then variant calling and independent filtering was performed using SAMtools mpileup v0.1.17[16] and GATK UnifiedGenotyper v1.3.31[17] and the callsets were merged. Exome variant profiles were filtered by standardised protocol using EVAR software tool vs 0.2.2 beta (www.exome.info)[11-13]. They were filtered by quality score using a standard cut-off value that implicates a base error rate < 0.1%, and we removed all variants occurring in dbSNP132, the 1000 Genomes and NHLBI Exome project with a minor allele frequency >0.1%. Remaining variants were filtered for affecting protein coding regions and known splice sites (up to 15 basepairs away from the exon-intron boundary), and synonymous changes were removed. Variants were then filtered against an in-house database to remove any occurring with a frequency >0.1%. Remaining biallelic variants were prioritised according to their presence in cilia proteome databases and mutation type. Coverage of known disease causing genes was manually analysed using Integrated Genome Viewer. Copy number variant analysis was performed using ExomeDepth[18]. Functional effects of variants were assessed using Polyphen2 and Mutationtaster.

Linkage analysis

Genome-wide homozygosity mapping was performed using GeneChip Human Mapping 250K NspI arrays (Affymetrix) on DNA samples from parents and 8 children from family UCL4 (I.1, I.2, II.1, II.2, II.3, II.4, II.5, II.7, II.8, II.9). Linkage data were generated by the Bioinformatics platform (Université Paris Descartes, Paris) and visualized using the interface created by the Bioinformatic platform. Linkage analysis was performed using MERLIN[19] considering a threshold of 1.0 and a minimum chromosomal region length of 2 Mb.

Antisense-morpholino knockdown in zebrafish

Zebrafish husbandry and morpholino techniques were performed according to standard procedures[20-23] with licenced approval from the Home Office (UK) under the Animal (Scientific Procedures) Act 1986. MO sequences were *p53* MO: 5'-GCGCCATTGCTTTGC-3'; control MO: 5'-TAGTGCAAAGCTTA-3'; *tctex1d2* MO1 (exon2-intron2 splice site): 5′-CTAAATCGTACTGTGCTGTTACCTT-3′; *tctex1d2* MO2 (intron2-exon3 splice site) 5'-CATGTCTGAGGAAGAGCAAATATAC-3'. *tctex1d2* morpholinos were designed by Genetools LCC on *ENSDART00000076424* (transcript zebrafish *tctex1d2-001*). MOs were dissolved in ddH2O and injections were performed at the 1-4 cell stage. Controls were injected with non-silencing control morpholino not targeting any known zebrafish gene to control for unspecific effects due to the injection and morpholino doses for sub-phenotypic co-injection experiments were balanced with this non-silencing control morpholino. To rule out phenotypic effects due to morpholino-dependant p53 activation, all embryos were co-injected with 5ng of p53 Morpholino[20]. Morpholino effects on transcript level were assessed by reverse transcription

Omniscript kit (Qiagen) using Trizol-Chloroform extracted total RNA obtained from 3 dpf embryos and primers listed in Supplementary Table 1. Light microscopy imaging was performed for whole mount embryos fixed in 4% PFA and mounted in methylcellulose, with cartilage stained using Alcian-Blue[20,22].

Immunofluorescence in *Chlamydomonas*

Cells were fixed with cold methanol and stained for immunofluorescence microscopy as adapted from Sanders and Salisbury[24]. Cells were allowed to adhere to the coverslips for about 3 minutes before fixation. Fixed and dried cells on coverslips were re-hydrated in PBS buffer for 1 hour and blocked in blocking buffer (5% BSA, 1% fish skin gelatin, 10% goat serum in 1×PBS) for 1 hour. Cells were incubated with the primary antibody diluted in blocking buffer overnight at 4°C. Incubation with the secondary antibody was done at room temperature for 2 hours. The coverslips were mounted onto the slides with ProLong antifade reagent (Molecular Probes, Eugene, OR). Images were acquired with an AxioCam camera, AxioVision 3.1 software, and an Axioskop 2 plus microscope (Zeiss). Images were processed using Adobe Photoshop (Adobe Systems Incorporated, San Jose, CA).

***Chlamydomonas* protein biochemistry**

To prepare *Chlamydomonas* whole cell lysates, cell pellets were re-suspended in water. An equal volume of SDS-PAGE sample loading buffer (50 mM Tris, pH 8, 160 mM DTT, 5 mM EDTA, 50% sucrose, 5% SDS, 0.04% Bromophenol Blue) was added to lyse the cells. When necessary, cells were passed 3-5 times through a 26-gauge needle to shear genomic DNA. To isolate flagella, cells were washed twice in 10 mM Hepes, re-suspended in HMS buffer (10 mM HEPES, pH 7.4, 5 mM $MgSO_4$, 4% sucrose). 2 ml of 25 mM dibucaine hydrochloride was added to 10 ml concentrated cells to deflagellate the cells. The flagella were separated from the cell bodies by centrifugation at 1800g for 5 minutes at 4°C. The supernatant containing flagella was collected, underlaid with 25% sucrose in 10 mM HEPES, pH 7.4, 5 mM $MgSO_4$ and further purified by centrifugation at 2400g for 10 minutes at 4°C to remove remaining cell bodies. The flagella were then harvested by centrifugation (30,000g, 4°C, 20 minutes) and re-suspended in HMDEK buffer (30 mM HEPES, pH 7.4, 5 mM $MgSO_4$, 1 mM DTT, 0.5 mM EGTA, 25 mM KCl). Flagella were treated with 0.1% NP-40 to yield the membrane-plus-matrix fractions. The flagellar membrane-plus-matrix fractions were layered on top of 12-ml 5-20% sucrose density gradients and centrifuged in a SWTi41 rotor at 36,000 rpm for 12 hours. Gradients were fractionated into 0.5-ml aliquots and subsequently analysed by SDS-PAGE and western blotting. For western blotting, the antibodies used are listed in Supplementary Table 6. When needed, protein samples were concentrated using Ultracel®-3k filters (Merck kGaA). Signals on blots were detected by use of x-ray film. Contrast was adjusted using Adobe Photoshop (Adobe Systems Inc.). Western blots were quantitated by comparing the intensity of a protein band in the mutant fraction to that of the same protein in a serial dilution of the wild-type fraction on the same western blot.

Proteomics in mammalian cells

HEK293T cells were transfected with constructs expressing SF-TAP-TCTEX1D2, or SF-TAP-RAF1 as a control, using polyethyleneimine (PEI, Polysciences), cultured for 48 hours and then lysed in lysis buffer containing 30 mM Tris–HCl (pH 7.4), 150 mM NaCl, 0.5% Nonidet-P40 (NP40), freshly supplemented with protease inhibitor cocktail (Roche), phosphatase inhibitor cocktail 2 (Sigma) and PhosphataseArrest III

(GBiosciences) for 20 minutes at 4°C. Streptavidin- and FLAG-based tandem affinity purification were performed using standard protocols[25,26]. 5% of the finale eluate was evaluated by PAGE followed by silver staining according to standard protocols, while the remaining 95% was subjected to protein precipitation with chloroform and methanol. Protein precipitates were subsequently subjected to mass spectrometry analysis and peptide identification[27]. Briefly, digested samples were separated on an UltiMate 3000 RSLCnano system, on-line coupled to a LTQ Orbitrap Velos (Thermo Fisher Scientific, Waltham, USA). All MS/MS samples were analyzed using Mascot (version 2.4, Matrix Science, Boston, MA, USA). Mascot was set up to search the human subset of the Swiss Prot database (Release 2013_12, 20274 entries), assuming trypsin as the digestion enzyme. Mascot was searched with a fragment ion mass tolerance of 0.5 Da and a parent ion tolerance of 10.0 PPM. Oxidation of methionine and was specified as variable modification, iodoacetamide derivative of cysteine as fixed. The Mascot results were loaded in Scaffold (version Scaffold_4, Proteome Software Inc., Portland, OR) to validate MS/MS based peptide and protein identifications. Peptide identifications were accepted if they could be established at >80.0% probability as specified by the Peptide Prophet algorithm[28]. Protein identifications were accepted if they could be established at >95.0% probability and contained at least 2 identified peptides. Protein probabilities were assigned using Protein Prophet[29]. Proteins containing similar peptides that could not be differentiated based on MS/MS analysis alone were grouped to satisfy the principles of parsimony.

Human immunoprecipitation protocol

HEK293T cells were transfected with the corresponding constructs using Lipofectamine 2000 (Life Technologies Ltd), according to the manufacturer’s protocol. Cells were harvested 48 hours after transfection in 500 ml of lysis buffer (1% NP40, 20 mM Tris-HCl pH 7.4, 50 mM NaCl + Roche Complete Protease inhibitor cocktail), homogenized with a 27G needle 10 times and then centrifuged at 4°C for 5min, 15800 g. Supernatant was collected and used for downstream applications or stored short-term at -20°C. 20 µl of Protein G Dynabeads were washed 3 times with 200 µl PBTw (PBS + 0.05% Tween-20), and then resuspended in 200 µl of PBTw and 1 µg of the corresponding antibody was added. The solution was rotated at RT for 90 mins, then the beads:antibody complexes were washes 3 times in 500 µl PBTw. After the last wash, complexes were resuspended in 200 µl IP buffer (0.5% NP40, 20 mM Tris-HCl pH 7.4, 50 mM NaCl + Roche Complete Protease inhibitor cocktail). Cell lysate was combined with resuspended beads:antibody complex, and IP buffer was added to a final volume of 1 ml. Mix was rotated overnight at 4°C. Beads were washed 3 times with 1 ml IP buffer, then resuspended in 50 ul of 1x Sample Buffer (5x sample buffer, 250 mM Tris-HCl pH 6.8, 10% SDS, 30% glycerol, 5% beta-mercaptoethanol, 0.02% bromophenol blue, diluted to 1x in lysis buffer). Samples were boiled for 5 min then loaded on acrylamide gels for western blot or stored short-term at -20°C.

Web resources

dbSNP: http://www.ncbi.nlm.nih.gov/projects/SNP/snp_summary.cgi?build_id=132
1000 Genomes: http://www.1000genomes.org/
ENSEMBL: http://www.ensembl.org
NHLBI exome Project: http//:evs.gs.washington.edu/EVS
Polyphen2: http://genetics.bwh.harvard.edu/pph2/2.
Mutationtaster: www.mutationtaster.org
IGV: www.broadinstitute.org/igv
BLAST: http://blast.ncbi.nlm.nih.gov/Blast.cgi

Supplementary References

1. Rompolas, P., Pedersen, L.B., Patel-King, R.S. & King, S.M. Chlamydomonas FAP133 is a dynein intermediate chain associated with the retrograde intraflagellar transport motor. *Journal of cell science* **120**, 3653-65 (2007).
2. Pazour, G.J., Dickert, B.L. & Witman, G.B. The DHC1b (DHC2) isoform of cytoplasmic dynein is required for flagellar assembly. *The Journal of cell biology* **144**, 473-81 (1999).
3. Hou, Y., Pazour, G.J. & Witman, G.B. A dynein light intermediate chain, D1bLIC, is required for retrograde intraflagellar transport. *Molecular biology of the cell* **15**, 4382-94 (2004).
4. Patel-King, R.S., Gilberti, R.M., Hom, E.F. & King, S.M. WD60/FAP163 is a dynein intermediate chain required for retrograde intraflagellar transport in cilia. *Molecular biology of the cell* **24**, 2668-77 (2013).
5. King, S.M. & Patel-King, R.S. Identification of a Ca(2+)-binding light chain within Chlamydomonas outer arm dynein. *Journal of cell science* **108 (Pt 12)**, 3757-64 (1995).
6. King, S.M. *et al.* Brain cytoplasmic and flagellar outer arm dyneins share a highly conserved Mr 8,000 light chain. *The Journal of biological chemistry* **271**, 19358-66 (1996).
7. DiBella, L.M., Smith, E.F., Patel-King, R.S., Wakabayashi, K. & King, S.M. A novel Tctex2-related light chain is required for stability of inner dynein arm I1 and motor function in the Chlamydomonas flagellum. *The Journal of biological chemistry* **279**, 21666-76 (2004).
8. DiBella, L.M., Sakato, M., Patel-King, R.S., Pazour, G.J. & King, S.M. The LC7 light chains of Chlamydomonas flagellar dyneins interact with components required for both motor assembly and regulation. *Mol Biol Cell* **15**, 4633-46 (2004).
9. LeDizet, M. & Piperno, G. The light chain p28 associates with a subset of inner dynein arm heavy chains in Chlamydomonas axonemes. *Molecular biology of the cell* **6**, 697-711 (1995).
10. Cole, D.G. *et al.* Chlamydomonas kinesin-II-dependent intraflagellar transport (IFT): IFT particles contain proteins required for ciliary assembly in Caenorhabditis elegans sensory neurons. *The Journal of cell biology* **141**, 993-1008 (1998).
11. Schmidts, M. *et al.* Exome sequencing identifies DYNC2H1 mutations as a common cause of asphyxiating thoracic dystrophy (Jeune syndrome) without major polydactyly, renal or retinal involvement. *Journal of medical genetics* **50**, 309-23 (2013).
12. Schmidts, M. *et al.* Combined NGS approaches identify mutations in the intraflagellar transport gene IFT140 in skeletal ciliopathies with early progressive kidney Disease. *Human mutation* **34**, 714-24 (2013).
13. Olbrich, H. *et al.* Recessive HYDIN mutations cause primary ciliary dyskinesia without randomization of left-right body asymmetry. *American journal of human genetics* **91**, 672-84 (2012).
14. Li, H. & Durbin, R. Fast and accurate long-read alignment with Burrows-Wheeler transform. *Bioinformatics* **26**, 589-95 (2010).
15. McKenna, A. *et al.* The Genome Analysis Toolkit: a MapReduce framework for analyzing next-generation DNA sequencing data. *Genome research* **20**, 1297-303 (2010).
16. Li, H. *et al.* The Sequence Alignment/Map format and SAMtools. *Bioinformatics* **25**, 2078-9 (2009).
17. DePristo, M.A. *et al.* A framework for variation discovery and genotyping using next-generation DNA sequencing data. *Nature genetics* **43**, 491-8 (2011).
18. Plagnol, V. *et al.* A robust model for read count data in exome sequencing experiments and implications for copy number variant calling. *Bioinformatics* **28**, 2747-54 (2012).
19. Abecasis, G.R., Cherny, S.S., Cookson, W.O. & Cardon, L.R. Merlin--rapid analysis of dense genetic maps using sparse gene flow trees. *Nature genetics* **30**, 97-101 (2002).
20. Rooryck, C. *et al.* Mutations in lectin complement pathway genes COLEC11 and MASP1 cause 3MC syndrome. *Nature genetics* **43**, 197-203 (2011).
21. Mitchison, H.M. *et al.* Mutations in axonemal dynein assembly factor DNAAF3 cause primary ciliary dyskinesia. *Nature genetics* **44**, 381-9, S1-2 (2012).

22. Halbritter, J. *et al.* Defects in the IFT-B component IFT172 cause Jeune and Mainzer-Saldino syndromes in humans. *American journal of human genetics* **93**, 915-25 (2013).
23. Westerfield, M. *The Zebrafish Book: a Guide for the Laboratory Use of the Zebrafish (Brachydanio rerio).* , (University of Oregon Press, USA, 2000).
24. Sanders, M.A. & Salisbury, J.L. Immunofluorescence microscopy of cilia and flagella. *Methods in cell biology* **47**, 163-9 (1995).
25. Gloeckner, C.J., Boldt, K., Schumacher, A., Roepman, R. & Ueffing, M. A novel tandem affinity purification strategy for the efficient isolation and characterisation of native protein complexes. *Proteomics* **7**, 4228-34 (2007).
26. Boldt, K., van Reeuwijk, J., Gloeckner, C.J., Ueffing, M. & Roepman, R. Tandem affinity purification of ciliopathy-associated protein complexes. *Methods in cell biology* **91**, 143-60 (2009).
27. Texier, Y. *et al.* Elution profile analysis of SDS-induced subcomplexes by quantitative mass spectrometry. *Molecular & cellular proteomics : MCP* **13**, 1382-91 (2014).
28. Keller, A., Nesvizhskii, A.I., Kolker, E. & Aebersold, R. Empirical statistical model to estimate the accuracy of peptide identifications made by MS/MS and database search. *Analytical chemistry* **74**, 5383-92 (2002).
29. Nesvizhskii, A.I., Keller, A., Kolker, E. & Aebersold, R. A statistical model for identifying proteins by tandem mass spectrometry. *Analytical chemistry* **75**, 4646-58 (2003).
